# 2,3-Dibutoxynaphthalene-based tetralactam macrocycles for recognizing precious metal chloride complexes

**DOI:** 10.3762/bjoc.15.146

**Published:** 2019-07-02

**Authors:** Li-Li Wang, Yi-Kuan Tu, Huan Yao, Wei Jiang

**Affiliations:** 1 Shenzhen Grubbs Institute and Department of Chemistry, Southern University of Science and Technology, Xueyuan Boulevard 1088, Shenzhen 518055, China

**Keywords:** host–guest chemistry, macrocycles, molecular recognition, precious metal chloride complexes

## Abstract

Two new tetralactam macrocycles with 2,3-dibutoxynaphthalene groups as sidewalls have been synthesized and characterized. The macrocycle containing isophthalamide bridges can bind square-planar chloride coordination complexes of gold(III), platinum(II), and palladium(II) in CDCl_3_, while the macrocycle with 2,6-pyridine dicarboxamide bridging units cannot. This may be due to the shrunken cavity caused by intramolecular hydrogen bonds in the latter tetralactam macrocycle. The binding of the isophthalamide-based macrocycle is mainly driven by hydrogen bonds and electrostatic interactions. This naphthalene-based macrocycle has similar binding affinities to all the three abovementioned precious metal chloride complexes. This is in contrast to the fact that the tetralactam macrocycle with anthracene as the sidewalls only show good binding affinities to AuCl_4_^−^. The superior binding to all three complexes may be due to the conformational diversity of the naphthalene-based macrocycle, which make it conformationally adaptive to maximize the binding affinities. In addition, the macrocycle shows fluorescent quenching when adding the chloride metal complexes in its solution and may be used as a fluorescent sensor for the detection of these coordination complexes.

## Introduction

**Scheme 1:**
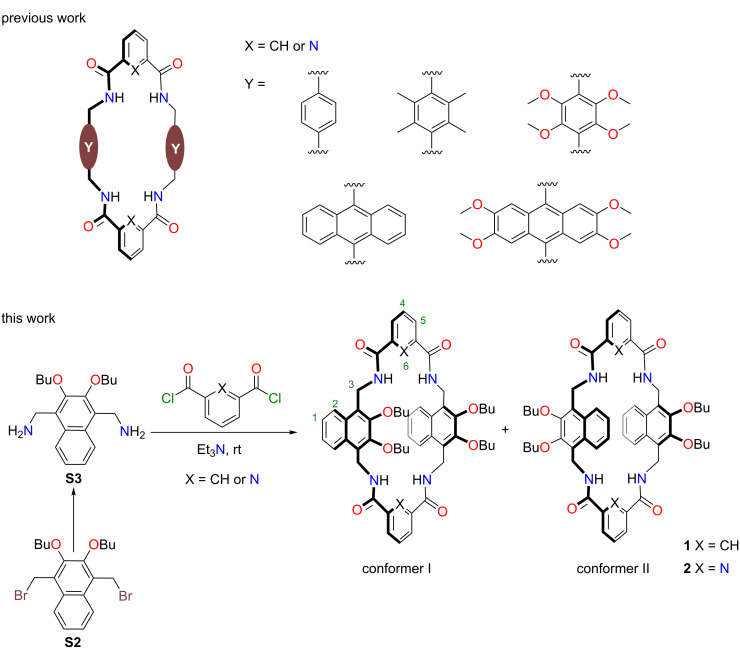
(a) Chemical structures of the reported tetralactam macrocycles with aromatic sidewalls; (b) synthetic procedure to 2,3-dibutoxynaphthalene-based tetralactam macrocycles. Numberings on the structures are used for the proton assignment in the NMR spectra, and the protons are designated as H1, H2, and so on.

Macrocyclic receptors are the major workhorses in supramolecular chemistry. Design and synthesis of new macrocyclic receptors with new properties is always attractive but is also challenging [1]. Tetralactam macrocycles with four amide NH residues have been studied for ca. three decades and used in a wide range of fields including molecular recognition, optical sensing, molecular machine etc. [2–13]. The amide N−H groups directed into the cavity could form hydrogen bonds with guests, constituting the main driving forces for molecular recognition. This class of macrocycles, in particular the ones with single arene as sidewalls, have found wide applications in diverse fields: Leigh and co-workers reported many interlocked structures and molecular machines with tetralactam macrocycles [14–16]. The Smith group has used tetralactam macrocycles to encapsulate and stabilize deep-red fluorescent squaraine dyes for biomedical applications [17–20]. By introducing water-soluble groups to tetralactam macrocycles, Davis and co-workers realized selective recognition of glucose in water [21–23]. However, the aromatic sidewalls of these tetralactams are currently limited to benzene, anthracene and their derivatives (Scheme 1). The extension of the sidewalls to naphthalenes should be an important supplement to the previous works, and may even lead to interesting new binding properties.

During the last six years, our group have developed a series of naphthol-based macrocyclic receptors [24–29]. Of them, oxatub[4]arene [30–31] and zorb[4]arene [32–33] show multiple conformations due to the flipping of naphthalenes and thus resulted in a rather wide guest scope. Therefore, we wondered whether multiple conformations would be observed by incorporating the 2,3-dialkoxynaphthalene units into tetralactam macrocycles as the sidewalls. How would this affect their binding affinity and selectivity of the resulting macrocycles?

Herein, we describe a pair of new tetralactam macrocycles with 2,3-dibutoxynaphthalene as the sidewalls. The tetralactam macrocycle with 2,6-pyridine dicarboxamide cannot bind square-planar chloride coordination complexes of gold(III), platinum(II), and palladium(II) in CDCl_3_, while the macrocycle containing isophthalamide bridging units bind these complexes with decent binding affinities. This macrocycle shows similar binding affinity to chloride, weaker affinity to the chloride complex of gold(III), and much stronger affinities to the chloride complexes of platinum(II) and palladium(II), when compared to the tetralactam macrocycle with anthracene sidewalls [34]. The difference in binding affinities and selectivity may be partially due to the conformational diversity in the tetralactam macrocycles with 2,3-dibutoxynaphthalene as the sidewalls. Moreover, they are fluorescent and could in principle be used as a fluorescent sensor to these precious metal chloride complexes.

## Results and Discussion

Synthetic procedures for the preparation of macrocycles **1** and **2** are shown in Scheme 1. The diamine **S3** was obtained by reacting the dibromide **S2** [32] with hexamethylenetetramine and then treating the product with acid and base. The butoxy groups were introduced to positioning the aminomethyl groups. Under pseudo-high dilution conditions, the diamine **S3** and the acid chlorides were added dropwise through a syringe pump to a flask containing Et_3_N and CH_2_Cl_2_ at room temperature. The tetralactam macrocycles **1** and **2** were isolated with yields of 22% and 19%, respectively.

**Figure 1:**
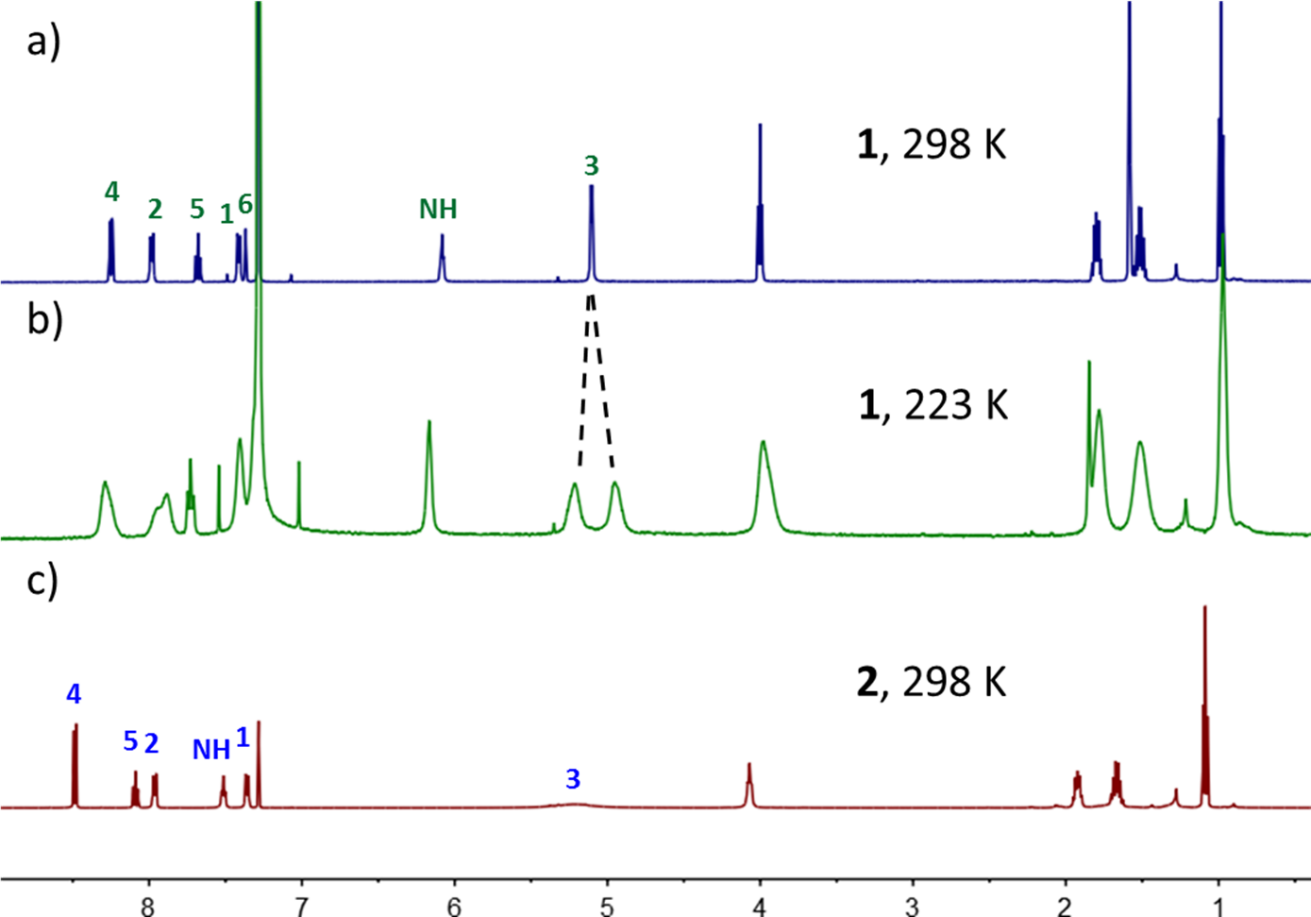
^1^H NMR spectra (500 MHz, CDCl3) of a) **1** at 298 K, b) **1** at 223 K, and c) **2** at 298 K.

Electrospray ionization (ESI) mass spectra support the isolated products to be the [2 + 2] macrocycles (see Supporting Information File 1). As shown in Figure 1, the ^1^H NMR spectra are consistent with high-symmetry structures. There are slight differences in the ^1^H NMR spectra of the two macrocycles. In particular, the NH protons of **2** (7.51 ppm) are located more downfield than that of **1** (6.08 ppm). This should be caused by the intramolecular hydrogen bonds with pyridine nitrogen atoms in **2** [35–38]. These hydrogen bonds make macrocycles **2** more pre-organized. In addition, there exist two conformations for both macrocycles **1** and **2** because the two 2,3-dibutoxynaphthalene moieties can be aligned either in parallel or antiparallel orientation (Scheme 1). These two conformations have different cavities defined by the parallel or antiparallel orientations of the two 2,3-dibutoxynaphthalene moieties; but the same number of peaks in NMR spectra should be observed. Nevertheless, only one set of signals are observed, suggesting that either only one conformation is predominant in solution or the conformational interconversion is fast at the NMR timescale. H3 is diastereotopic and could be split when the conformational interconversion is slow on the NMR timescale, as observed for oxatub[4]arene [30] and zorb[4]arene [32]. When lowering the temperature to 223 K, H3 of **1** was split to two peaks while other peaks become broadened (Figure 1b). This suggests that the conformational interconversion is slow at this temperature and only one conformer is predominant in the solution at 298 K. However, the exact conformer cannot be assigned because the two conformers have the same number of peaks. In contrast, H3 of **2** are broadened even at room temperature, which may be caused by intramolecular hydrogen bonds, but the conformational interconversion should be fast as well for macrocycle **2** at room temperature, which is supported by only one set of sharp signals for the aromatic protons and the non-split and sharp signals of diastereotopic protons at the butyl methylene group next to the oxygen atoms.

**Figure 2:**
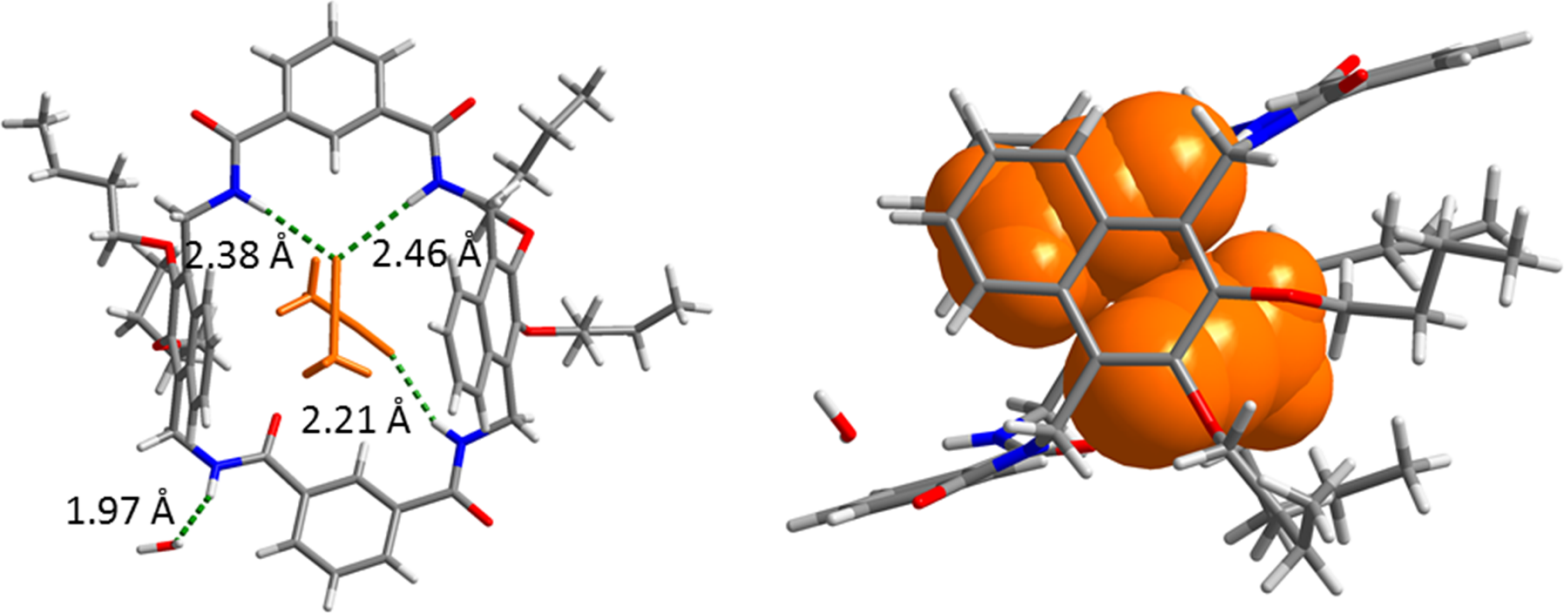
Two different views of the X-ray single crystal structure of **1** obtained from its CH_3_CN solution.

A single crystal (Figure 2) of **1**, which was suitable for X-ray crystallography, was obtained by slow evaporation of its solution in CH_3_CN. The crystal structure clearly shows that **1** adopts a flattened chair conformation with the two naphthalenes in parallel orientation. Three of the four NH protons are directed into the cavity, and the fourth one flipped outward and forms a hydrogen bond with the oxygen atom of H_2_O (H···O distance: 1.97 Å). Two CH_3_CN molecules were trapped in the cavities by the amide groups through N–H···N hydrogen bonds (H···N distance: 2.21, 2.38 and 2.46 Å). C–H···π interactions (H···π distance: 2.75 and 2.83 Å) between the methyl group of CH_3_CN and the naphthalene panels of the host are also detected. This suggests that the 2.3-dibutoxynaphthalene tetralactam macrocycle, just like other tetralactam macrocycles, may use hydrogen bonds to recognize guests with hydrogen-bonding acceptors.

**Figure 3:**
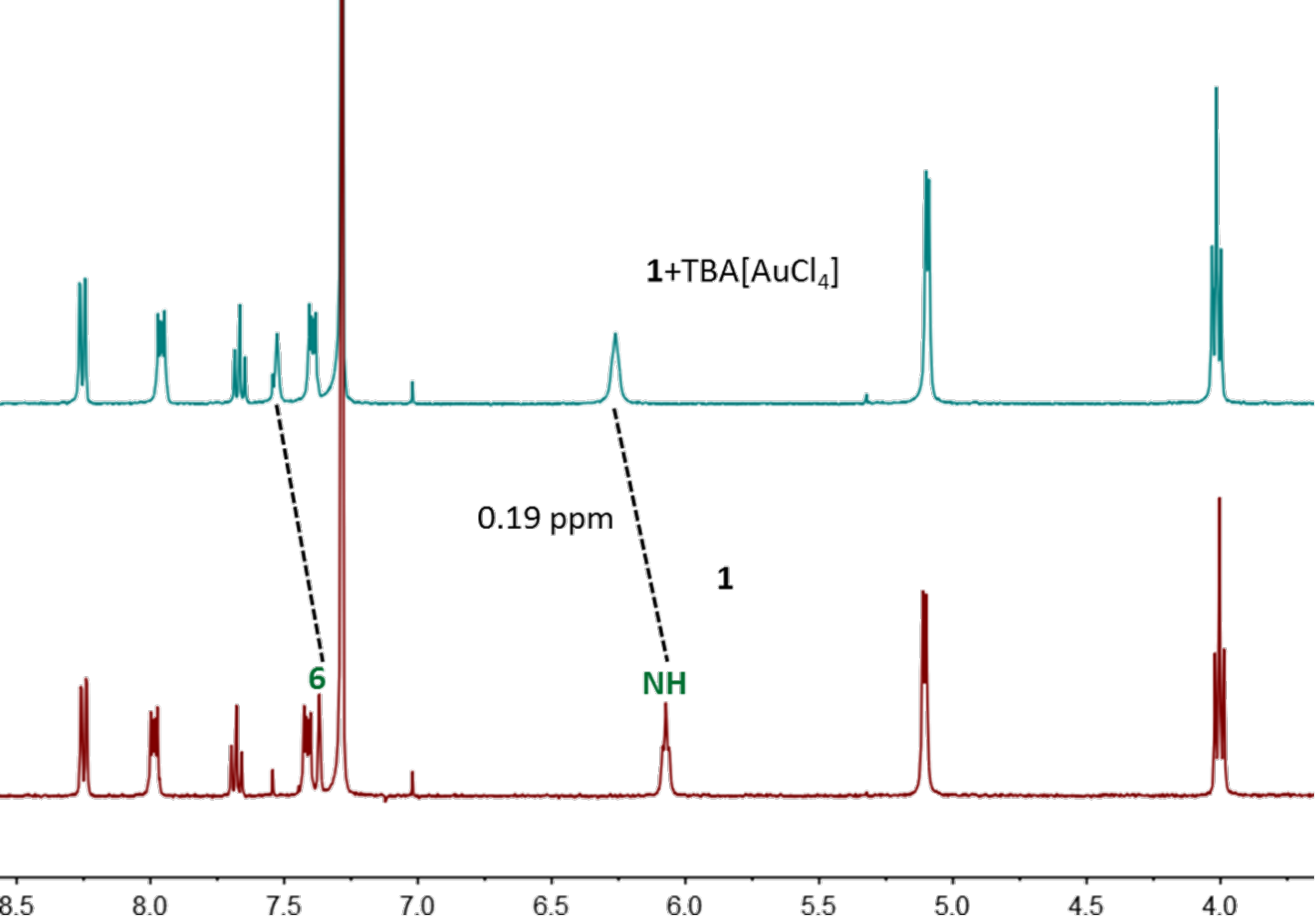
Partial ^1^H NMR spectra (500 MHz, CDCl_3_, 0.5 mM, 298 K) of **1** and the equimolar mixture with TBA[AuCl_4_].

Precious metal chloride complexes were found to be complexed by **1**. Obvious chemical shifts in the ^1^H NMR spectra (Figure 3) were observed for H6 and amide NH protons when adding one equivalent of the tetrabutylammonium (TBA^+^) salt of AuCl_4_^−^ to the solution of **1** in CDCl_3_. H6 and NH in the complex undergo downfield shifts when compared with free **1**, suggesting that these protons are involved in hydrogen bonds to the guest.

**Figure 4:**
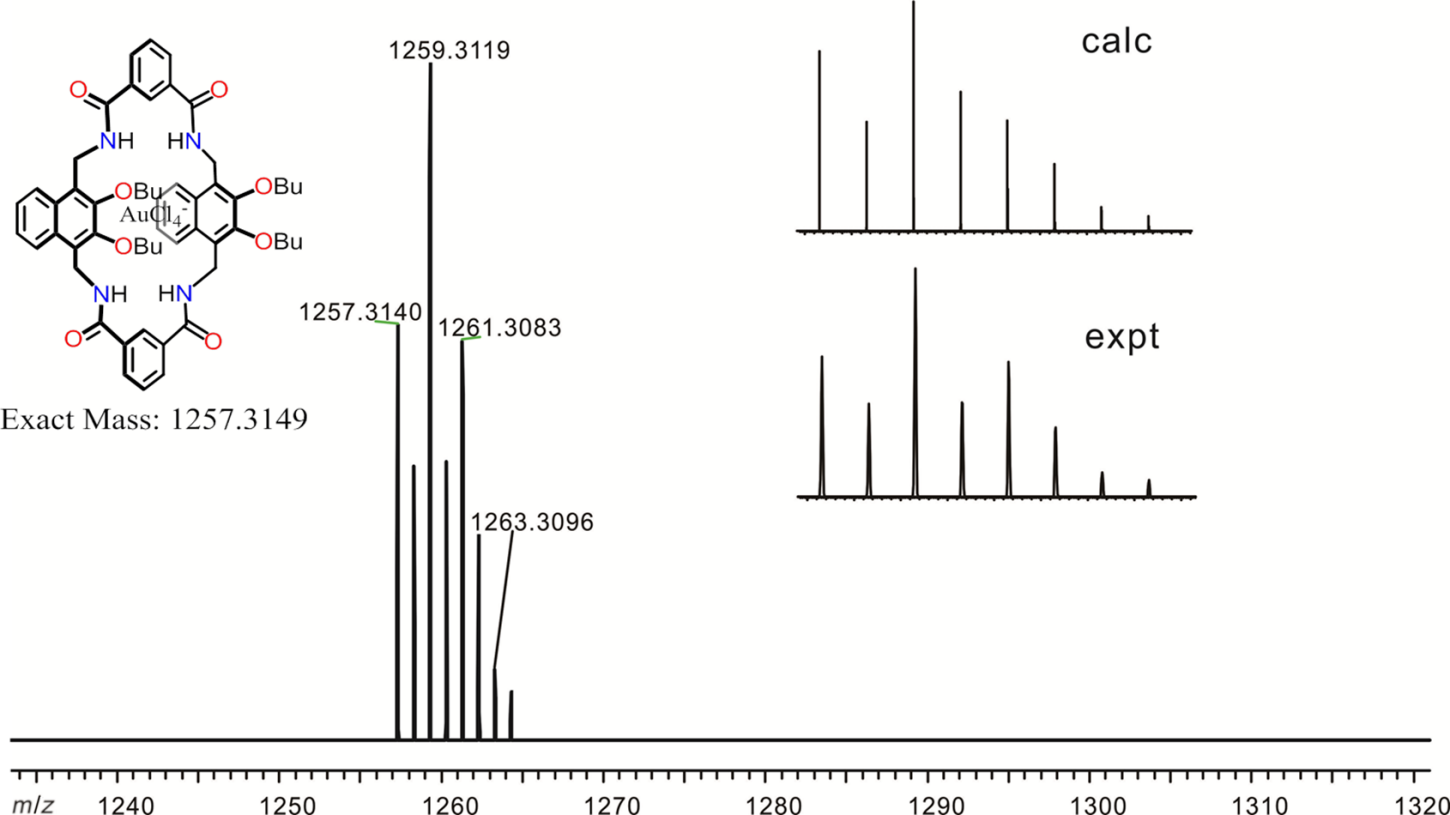
ESI mass spectrum of complex AuCl_4_^−^@**1**.

Job’s plot (Figures S1 and S2, Supporting Information File 1) indicates a 1:1 binding stoichiometry, which is further supported by the ESI mass spectrum (Figure 4). The peak at *m*/*z* 1257.3140 was assigned to AuCl_4_^−^@**1**. The experimental isotopic pattern is consistent with the calculated one. In addition, precious metal chloride complexes PdCl_4_^2−^ and PtCl_4_^2−^ with TBA^+^ as counter ion can be bound by **1** as well (Figures S3 and S4, Supporting Information File 1). The binding stoichiometry was also determined to be 1:1 by Job’s plots (Figures S5–S8, Supporting Information File 1). The chemical shift of NH protons of **1** upon addition of one equivalent of (TBA)_2_[PtCl_4_] or (TBA)_2_[PdCl_4_] (Figures S3 and S4, Supporting Information File 1) is smaller than that in the presence of one equivalent of TBA[AuCl_4_] (Figure 3), although they have rather similar binding affinities. This may be due to a different extent hydrogen bonding is involved in diverse complexes and other non-covalent interactions may contribute more in the cases of (TBA)_2_[PtCl_4_] or (TBA)_2_[PdCl_4_].

Binding constants of **1** to the precious metal chloride complexes were determined in CDCl_3_ by ^1^H NMR titrations (Figures S9−S14, Supporting Information File 1). The data are listed in Table 1. TBA[AuCl_4_] shows the highest association constant (*K*_a_) with a value of 265 ± 33 M^−1^. The *K*_a_ values for (TBA)_2_[PtCl_4_] and (TBA)_2_[PdCl_4_] were 189 ± 36 M^−1^ and 198 ± 15 M^−1^, respectively. It should be noted that PdCl_4_^2−^ is relatively labile at high concentrations and is in equilibrium with the palladate dimer (Pd_2_Cl_6_^2−^) and chloride ions [32,39]. The formation of Pd_2_Cl_6_^2−^ was supported by an ESI mass spectrum (Figure S15, Supporting Information File 1). Therefore, the association constant for (TBA)_2_[PdCl_4_] may, in fact, be caused by the mixture of (TBA)_2_[PdCl_4_], TBACl, and (TBA)_2_[Pd_2_Cl_6_]. Tetralactam macrocycles are known to bind chloride ions as well [4,40]. Indeed, **1** is able to bind TBACl with *K*_a_ = 90 ± 13 M^−1^ (Figures S16−S18, Supporting Information File 1). It is interesting that the NH peak broadens and disappears through the addition of TBACl (Figure S19, Supporting Information File 1) and the underlying reason is still unknown. A similar phenomenon was observed for other tetralactam macrocycles [41].

**Table 1:** Association constants *K*_a_ (M^−1^) of **1** binding to different guests in CDCl_3_ at 298 K determined by ^1^H NMR titrations.

guest	TBA[AuCl_4_]	(TBA)_2_[PtCl_4_]	(TBA)_2_[PdCl_4_]	TBACl

*K*_a_	265 ± 33	189 ± 36	198 ± 15	90 ± 13

These binding affinities are lower than those of a positively-charged macrocycle [42], but are comparable or superior when compared to other tetralactam macrocycles. With respect to the association constants to TBA[AuCl_4_] and TBACl, **1** is comparable to the tetralactam macrocycles with anthracene as the sidewalls [34], but is significantly weaker than the one with tetramethylphenyl sidewalls [26]. However, **1** (189 M^−1^) is superior to these two tetralactam macrocycles (no binding, 20 M^−1^, respectively) [26] in terms of the binding to (TBA)_2_[PtCl_4_]. This may be due to the existence of two conformations of **1**, which is conformationally adaptive to the guests. That is, the guest can select the better conformation to maximize the binding affinities, as oxatub[4]arene and zorb[4]arene do [30,32].

In contrast, the addition of these precious metal chloride complexes does not induce any change in the ^1^H NMR spectrum of **2** (Figure S20, Supporting Information File 1), suggesting **2** not to be a binder of these guests. This is surprising! The only difference between **1** and **2** is that a pyridine moiety is used in **2** in contrast to phenyl group in **1**. This leads to further hydrogen bonds between the amide NH protons and the pyridine nitrogen atoms in **2**, which pre-organizes the cavity for molecular recognition. How would this affect the binding affinity of **2**? To answer this question, molecular modelling was performed.

**Figure 5:**
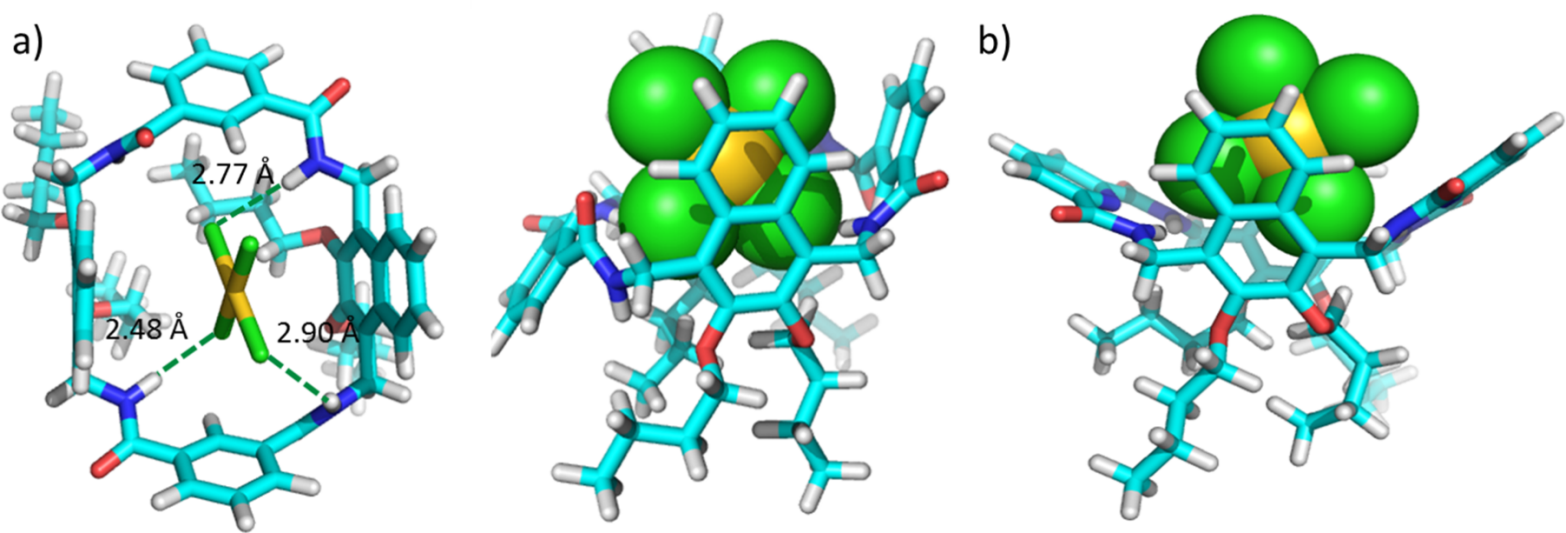
Energy-minimized structure of a) AuCl_4_^−^@**1** and b) AuCl_4_^−^@**2** at the level of theory of PM3 by using Spartan ’14 (Wavefunction, Inc.).

Only conformer I of **1** was used for the molecular modelling but both conformers are possible. As shown in Figure 5a, AuCl_4_^−^ can be well accommodated in the cavity of **1**. AuCl_4_^−^ is sandwiched by the two 2,3-dibutoxynaphthalene moieties, and the electron-poor gold centre of AuCl_4_^−^ may interact with the electron-rich naphthalenes through electrostatic interactions. In addition, hydrogen bonds are detected between the amide protons and H6 of **1** and the chloride ions of the guest (H···Cl distance: 2.48, 2.77 and 2.90 Å). Consequently, the electrostatic interaction and hydrogen bonds are the main driving forces for the host–guest complex. It is noted that the four amide protons are significantly distorted to accommodate AuCl_4_^−^ in the cavity of **1**. In contrast, AuCl_4_^−^ cannot be encapsulated in the cavity of **2** (Figure 5b). Hydrogen bonds between the pyridine moiety and adjacent amides significantly shrink the cavity [43], and thus the cavity cannot be distorted to fit AuCl_4_^−^. Otherwise, a high enthalpic penalty would be paid by destroying the intramolecular hydrogen bonds. This may explain why **2** is not a binder for these precious metal chloride complexes.

**Figure 6:**
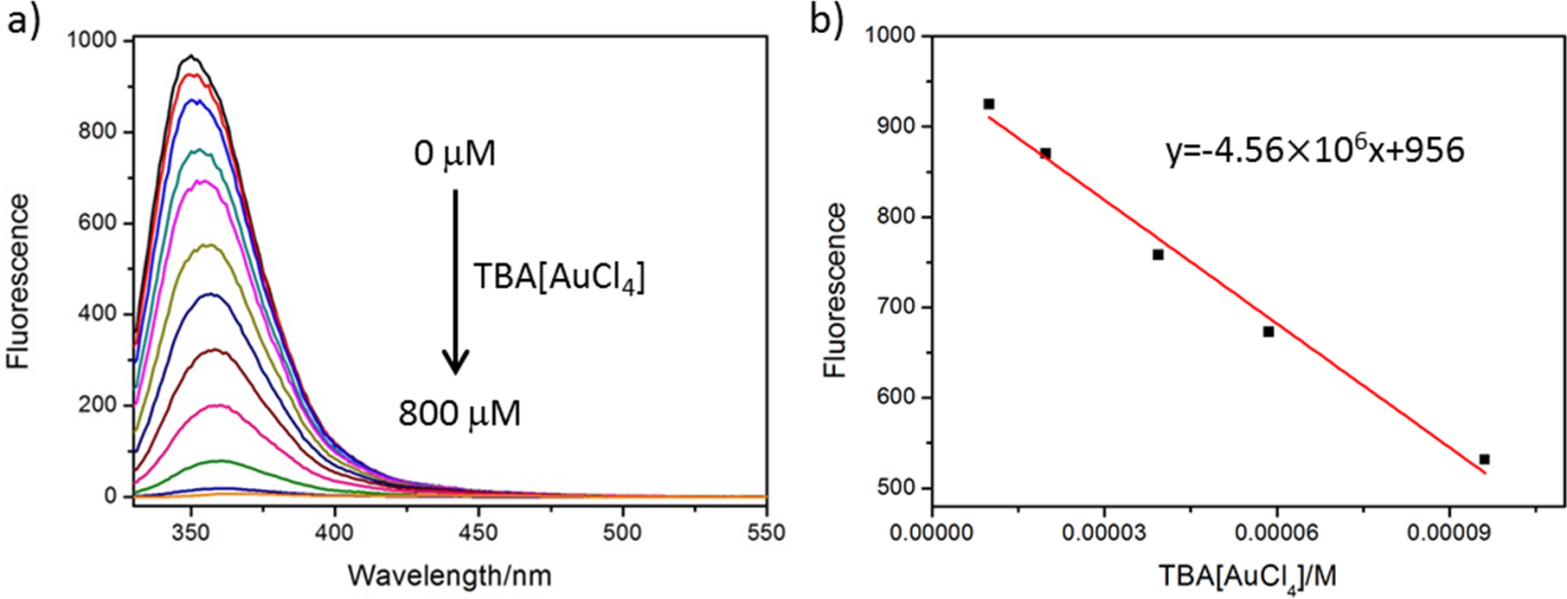
a) Fluorescence emission spectra of **1** (20 µM) upon addition of different amounts of TBA[AuCl_4_] (concentration range of 0.0−800 µM) and then recorded in dichloromethane at room temperature (λ_ex_ = 310 nm). b) Plot of fluorescence intensity versus TBA[AuCl_4_] concentration (10−96 µM).

Macrocycle **1** is fluorescent because of the 2,3-dibutoxynaphthalene sidewalls and may be used as a fluorescent sensor for these precious metal chloride complexes. The UV–vis absorption spectrum was shown in Figure S21 (Supporting Information File 1), and the absorption maxima are located at 241 nm and 290 nm. However, the optimal excitation wavelength is at 310 nm with a quantum yield of 6.6% in dichloromethane. The emission maximum of **1** is located at 350 nm (Figure S22, Supporting Information File 1). It is known that the binding of metal ions could cause fluorescence quenching through photo-induced electron transfer [44]. Indeed, the fluorescence intensity of **1** is quenched gradually upon addition of TBA[AuCl_4_] (Figure 6a). A linear relationship between fluorescent intensity and the concentrations of TBA[AuCl_4_] was observed at the concentration range of 20–96 μM, giving a detection limit of 0.61 μM for TBA[AuCl_4_] according to standard methods [45] (Figure 6b). This is similar for (TBA)_2_[PtCl_4_] within a linear concentration range of 20–970 μM and a detection limit of 3.9 μM (Figure S23, Supporting Information File 1). For (TBA)_2_[PdCl_4_], the fluorescence intensity was enhanced at the beginning (Figure S24, Supporting Information File 1), and then quenched with increasing concentrations of (TBA)_2_[PdCl_4_] (Figure S25a, Supporting Information File 1). This may be due to the dissociated TBACl which can induce the fluorescent enhancement of **1** (Figure S26, Supporting Information File 1). A linear concentration range from 60 to 1270 μM provides a detection limit of 7.4 μM for (TBA)_2_[PdCl_4_] (Figure S25b, Supporting Information File 1).

## Conclusion

In summary, we reported the synthesis of two new tetralactam macrocycles with 2,3-dibutoxynaphthalenes as the sidewalls. The macrocycle with isophthalamide as the linkers is able to bind chloride ions and precious metal chloride complexes in nonpolar solvent, while the macrocycle with 2,6-pyridine dicarboxamide bridging units cannot. This may be due to the shrunken cavity caused by intramolecular hydrogen bonds in the latter tetralactam macrocycle. The binding driving forces of the isophthalamide-based macrocycle are mainly from hydrogen bonds and electrostatic interactions between the naphthalenes and the metal centre. Moreover, this naphthalene-based tetralactam macrocycle is generally a good binder for TBA(AuCl_4_], (TBA)_2_[PtCl_4_], and (TBA)_2_[PdCl_4_] complexes, while the tetralactam macrocycles with anthracene and tetramethylphenyl sidewalls [26] shows rather poor binding affinities to (TBA)_2_[PtCl_4_]. This may be due to the possibility of the naphthalene-based macrocycle to adapt its conformation according to the need of the guests to maximize the binding affinities through naphthalene flipping. In addition, this macrocycle is fluorescent and can, in principle, be used as a sensor for the detection of precious metal chloride complexes. These new tetralactam macrocycles with 2,3-dialkoxynaphthalene groups as sidewalls provide a new supplement to the tetralactam macrocycle family and may find further applications in molecular recognition and molecular machines.

## Supporting Information

File 1 Experimental procedures, NMR and mass spectra, determination of association constants and X-ray single crystal data.
