# Supplementary material for: 2,3-Dibutoxynaphthalene-based tetralactam macrocycles for recognizing precious metal chloride complexes
Source: Beilstein J Org Chem. 2019 Jul 2;15:1460–7. doi: 10.3762/bjoc.15.146 (PMC6633862; doi:10.3762/bjoc.15.146)
Supplement: File 1 — Experimental procedures, NMR and mass spectra, determination of association constants and X-ray single crystal data. [file Beilstein_J_Org_Chem-15-1460-s001.pdf]

BEILSTEIN JOURNAL OF ORGANIC CHEMISTRY

Supporting Information

for

2,3-Dibutoxynaphthalene-based tetralactam macrocycles for recognizing precious metal chloride complexes

Li-Li Wang, Yi-Kuan Tu, Huan Yao and Wei Jiang

Beilstein J. Org. Chem. **2019,** *15,* 1460–1467. doi:10.3762/bjoc.15.146

Experimental procedures, NMR and mass spectra, determination of association constants and X-ray single crystal data

License and Terms: This is a supporting information file under the terms of the Creative Commons Attribution License (http://creativecommons.org/licenses/by/4.0). Please note that the reuse, redistribution and reproduction in particular requires that the authors and source are credited.
The license is subject to the *Beilstein Journal of Organic Chemistry* terms and conditions: (https://www.beilstein-journals.org/bjoc)

Table of Contents

1. Experimental section S2
2. Synthetic procedures S3
3. ^{1}H NMR spectra and Job's plots of the complexes S9
4. Binding constants determined by NMR titrations S13
5. ^{1}H NMR spectra of **2** in the presence of guests S18
6. Optical spectra of host–guest complexes S19
7. X-ray single crystal data S22
8. References S22

1. Experimental section

General method. All the reagents involved in this research were commercially available and used without further purification unless otherwise noted. Solvents were either employed as purchased or dried prior to use by standard laboratory procedures. ^1H NMR spectra were recorded on Bruker Avance-400 (500) spectrometers. All chemical shifts are reported in ppm with residual solvents or TMS (tetramethylsilane) as the internal standards. Electrospray-ionization time-of-flight high-resolution mass spectrometry (ESI-TOF-HRMS) experiments were conducted on an applied biosystems Elite ESI-QqTOF mass spectrometry system. Fluorescence spectra were obtained on a Shimadzu RF-5301pc spectrometer. UV–vis absorption spectra were obtained on a Hitachi U-2600 UV–vis spectrophotometer. Absolute luminescent quantum yields were recorded with Hamamatsu absolute FL quantum yield spectrometer C11347. The energy-minimized structure was performed using the PM3 Hamiltonian implemented in the Spartan 14 program. The initial structure of **1** was extracted from its crystal structure. Synthesis of compounds **S1** and **S2** were reported earlier [1].

NMR titration. NMR titration experiments were conducted in $CDCl_3$ at 298 K. Aliquots from a stock solution containing the appropriate tetrabutylammonium (TBA^+) salt were added sequentially to an NMR tube containing the macrocyclic host and one ^1H NMR spectrum was acquired after each addition. The titration isotherms were fitted to a 1:1 binding model using Thordarson's equation shown below. A standard non-linear squares method within OriginPro 8 software was used for determining binding constant (K_a). δ_0 is the NMR resonance of the host before the guest is added.

$$\delta=\delta_0+\Delta\delta\ (0.5/[H]_0)\ ([G]+[H]_0+1/K_a\ -(([G]+[H]_0+1/K_a)\char`\^2-4[H]_0[G])\char`\^0.5)$$

2. Synthetic procedures

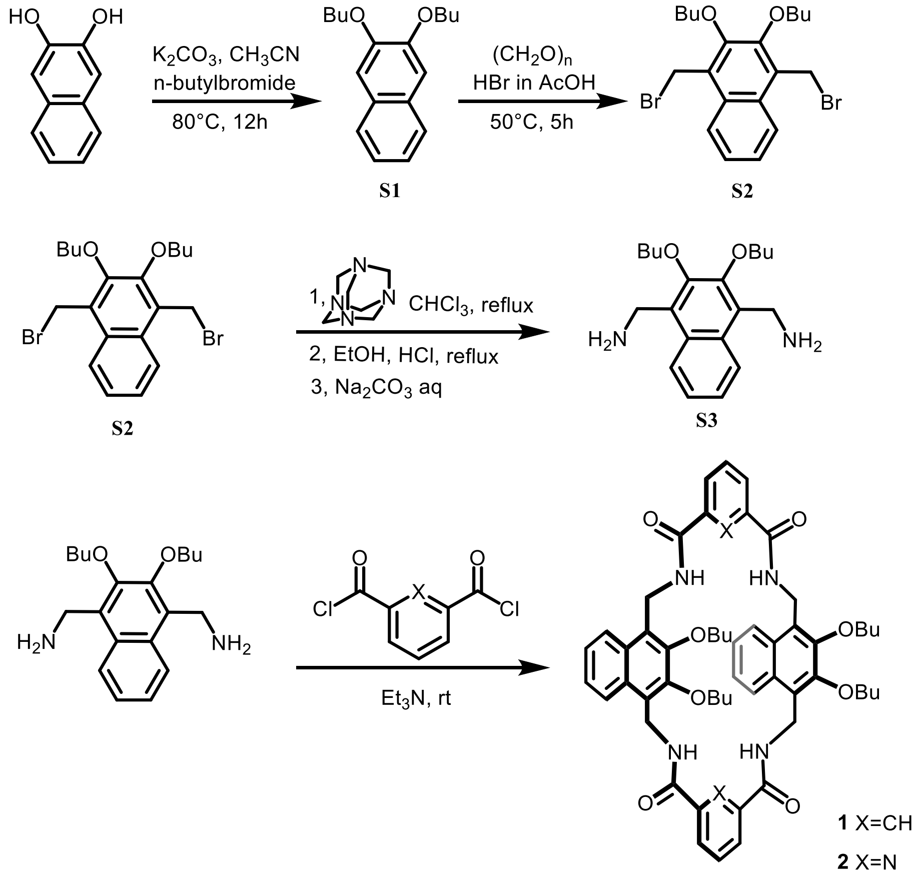

Scheme S1: Syntheses route of **1** and **2**.

Compound S3: S2 (4.58 g, 10 mmol) and hexamethylenetetramine (4.2 g, 30 mmol) were suspended in chloroform (300 mL), and the reaction mixture was refluxed for 48 h. The mixture was allowed to cool and the precipitate was collected by filtration. The solid was air-dried then suspended in ethanol (250 mL). Concentrated aq. HCl (50 mL) was added and the mixture was refluxed for 48 h. The mixture was cooled to 0 °C, and the resulting precipitate was collected by filtration and washed with cold ethanol. The solid was suspended in Na_2CO_3 aq. (2 M, 200 mL) with stirring. Chloroform (200 mL) was added to extract the resulting amine. The aqueous phrase was extracted with additional chloroform (100 mL). The organic phases were combined and dried over Na_2SO_4. The solvent was removed to give **S3** (2.45 g, 74%) as a yellow liquid. δ [ppm]

= ^{1}H NMR (500 MHz, $CDCl_3$) δ: 8.09 (dd, J = 6.5, 3.3 Hz, 2H), 7.51 (dd, J = 6.5, 3.3 Hz, 2H), 4.31 (s, 4H), 4.08 (t, J = 6.7 Hz, 4H), 1.90 - 1.78 (m, 4H), 1.59 - 1.54 (m, 4H, overlap with HOD signal), 1.04 (t, J = 7.4 Hz, 6H). ^{13}C NMR (126 MHz, $CDCl_3$) δ: 149.29, 130.91, 129.78, 125.33, 124.04, 74.17, 36.80, 32.61, 19.44, 14.07.

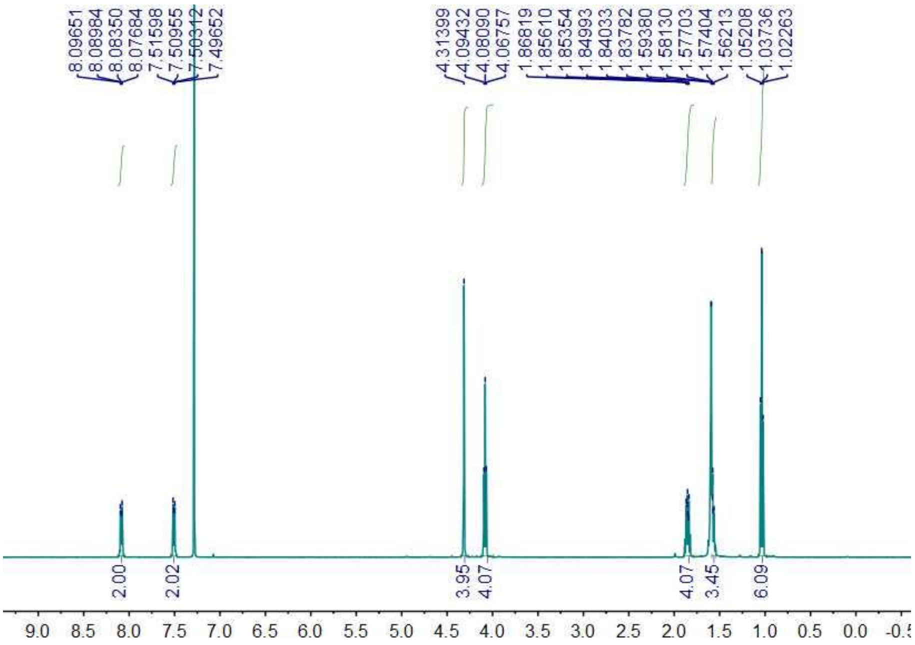

^{1}H NMR spectrum (500 MHz, $CDCl_3$, 298 K) of compound **S3**

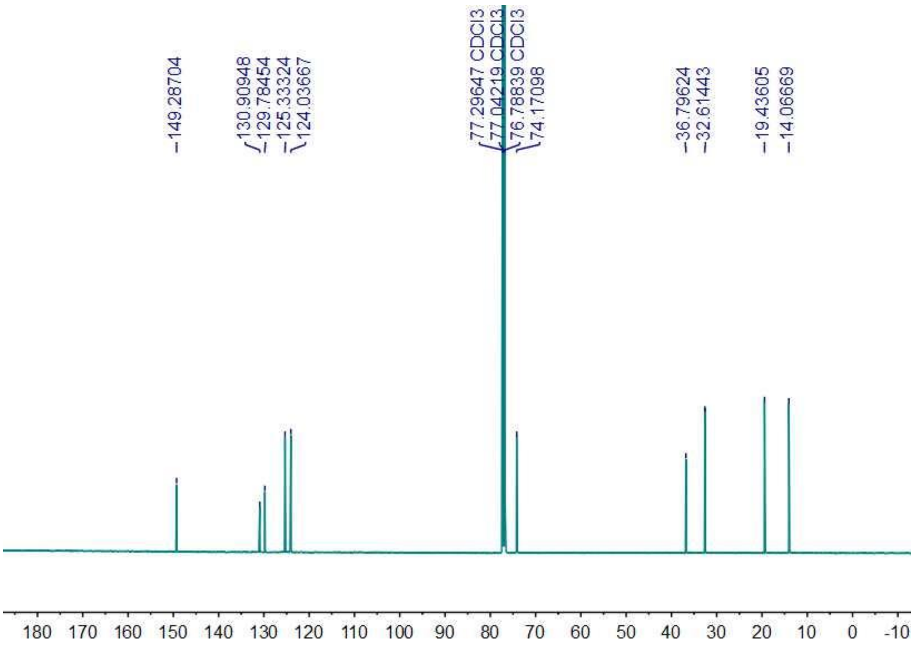

^{13}C NMR spectrum (125 MHz, $CDCl_3$, 298 K) of compound **S3**

Compound 1 and 2: The diamine **S3** (660 mg, 2.0 mmol) was dissolved in 60 mL dry dichloromethane (DCM) (flask A). In a separate flask (B), clear solutions of the corresponding diacid dichloride (2.0 mmol) was dissolved in dry DCM (60 mL). In a third flask (C) was placed DCM (300 mL) and Hünig's base (3.4 mL, 20.0 mmol). By using a double syringe pump, the contents of flask A and flask B (separate syringes) were added dropwise to flask C over a period of 10 h at room temperature. The resulting mixture was stirred for another 6 h. Solvents were removed in vacuo, and the residue was dissolved in DCM (50 mL). The organic layer was washed with 10% aqueous HCl (50 mL) followed by sat. aqueous $NaHCO_3$ (100 mL) and dried over Na_2SO_4. Following removal of the solvent in vacuo, the residue was purified by column chromatography (CH_3OH/DCM = 1/100) to afford **1** or **2** as a white solid.

1. White solid, yield (163 mg, 22 %), m.p. =195 °C; ^{1}H NMR (500 MHz, $CDCl_3$) δ: 8.25 (d, J = 7.8 Hz, 2H), 7.98 (dd, J = 6.5, 3.4 Hz, 2H), 7.68 (t, J = 7.8 Hz, 1H), 7.41 (dd, J = 6.6, 3.2 Hz, 2H), 7.38 (s, 1H), 6.10 (t, J = 5.1 Hz, 2H), 5.10 (d, J = 4.9 Hz, 4H), 4.00 (t, J = 6.6 Hz, 4H), 1.80 (p, J = 6.9 Hz, 4H), 1.51 (h, J = 7.4 Hz, 4H), 0.98 (t, J = 7.3 Hz, 6H). ^{13}C NMR (126 MHz, $CDCl_3$) δ: 166.38, 150.69, 134.14, 132.13, 129.92, 129.83, 126.31, 125.85, 124.33, 121.61, 74.62, 35.45, 32.46, 19.36, 13.99. ESI-HRMS for $[M+H]^+$ calcd for $C_{56}H_{65}N_4O_8^+$, 921.4797; found 921.4784, error = -1.4 ppm.

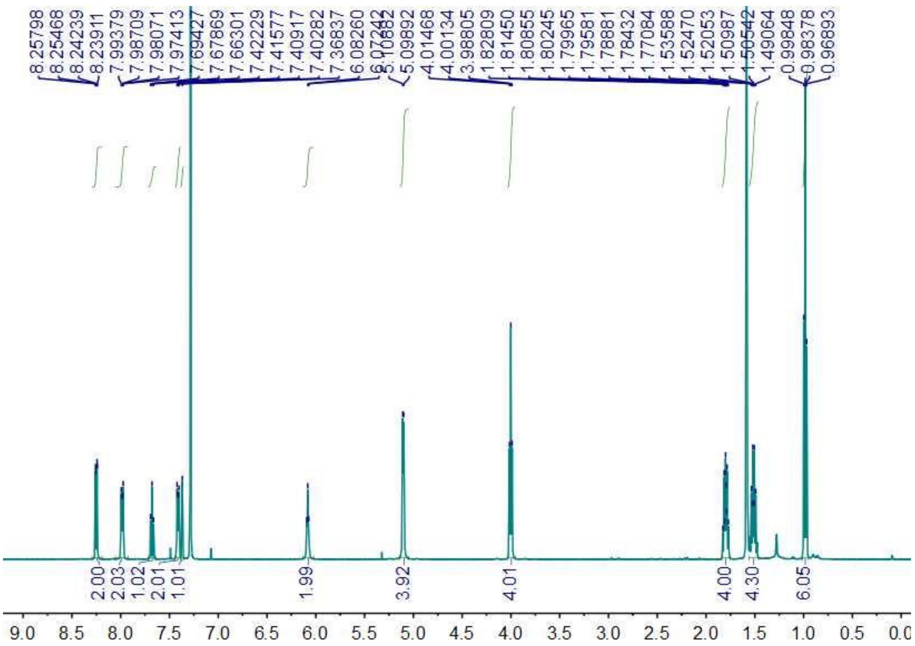

^{1}H NMR spectrum (500 MHz, $CDCl_3$, 298 K) of compound **1**

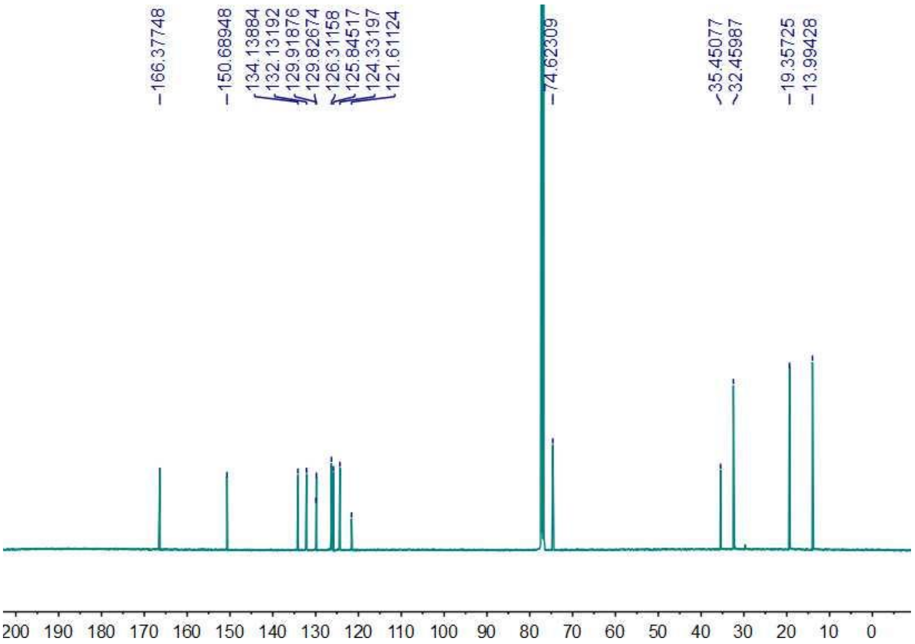

^{13}C NMR spectrum (125 MHz, $CDCl_3$, 298 K) of compound **1**

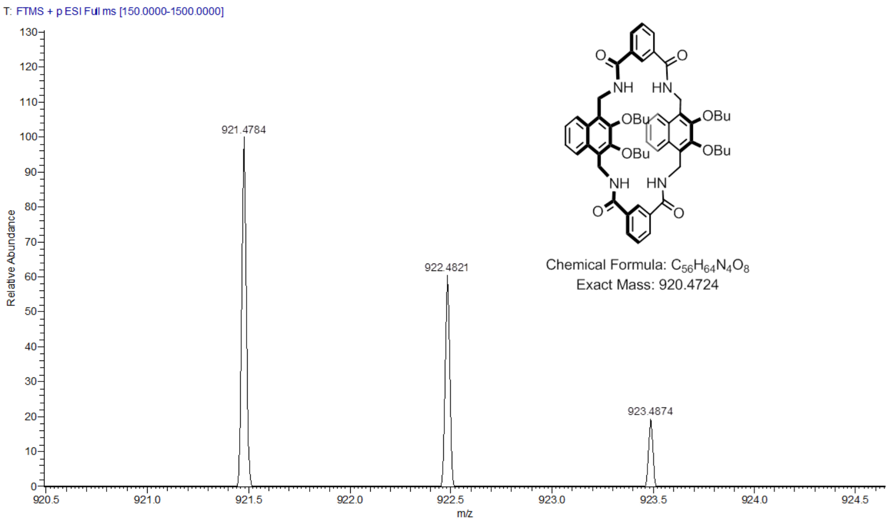

ESI mass spectrum of compound **1**

2. White solid, yield (141 mg, 19 %), m.p. = 260 °C; ^{1}H NMR (500 MHz, $CDCl_3$) δ: 8.48 (d, J = 7.9 Hz, 4H), 8.09 (t, J = 7.8 Hz, 2H), 7.96 (dd, J = 6.4, 3.3 Hz, 4H), 7.51 (t, J = 5.9 Hz, 4H), 7.36 (dd, J = 6.5, 3.2 Hz, 4H), 4.07 (t, J = 6.4 Hz, 8H), 2.01-1.85 (m, 8H), 1.67 (dt, J = 15.2, 7.4 Hz, 8H), 1.09 (t, J = 7.4 Hz, 12H).^{13}C NMR (126 MHz, $CDCl_3$) δ: 162.67, 150.50, 148.38, 139.01, 129.84, 126.32, 126.01, 125.66, 124.49, 74.81, 34.03, 32.59, 19.68, 14.04. ESI-HRMS for $[M+H]^+$ calcd for $C_{54}H_{63}N_6O_8^+$, 923.4702; found 923.4690, error = -1.3 ppm.

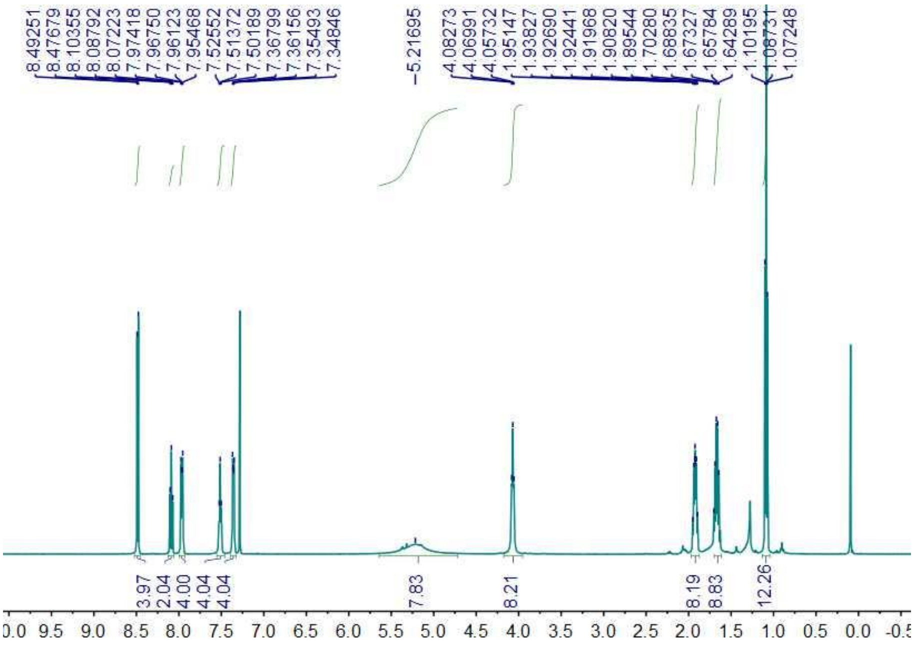

^{1}H NMR spectrum (500 MHz, $CDCl_3$, 298 K) of compound **2**

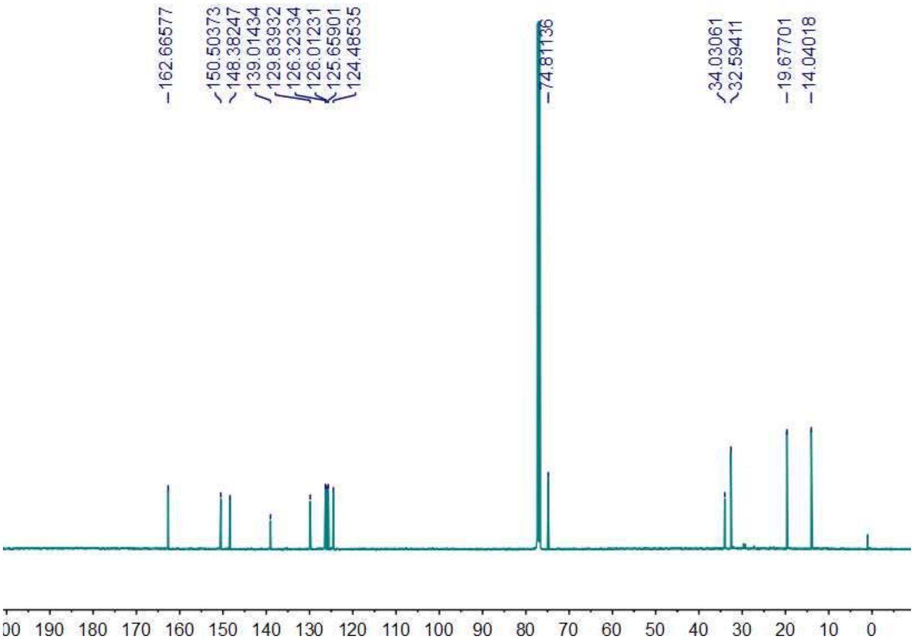

^{13}C NMR spectrum (125 MHz, $CDCl_3$, 298 K) of compound **2**

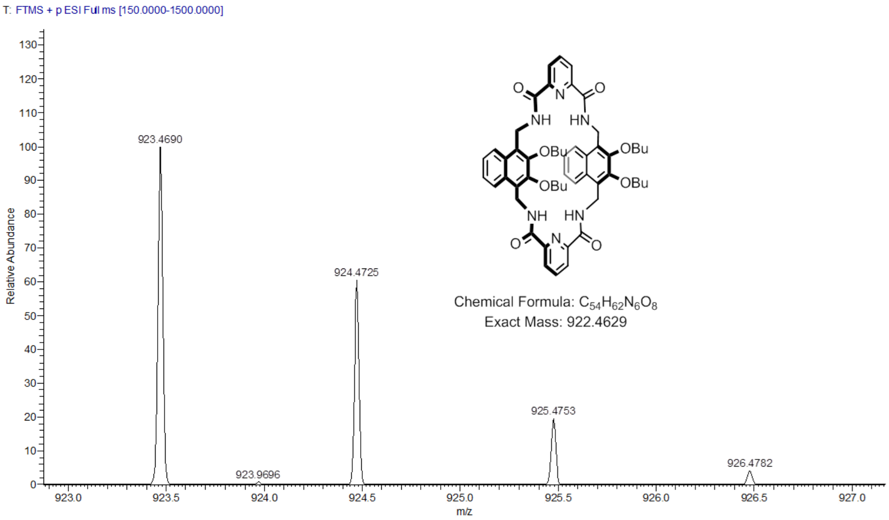

ESI mass spectrum of compound **2**

3. ^{1}H NMR spectra and Job's plots of the complexes

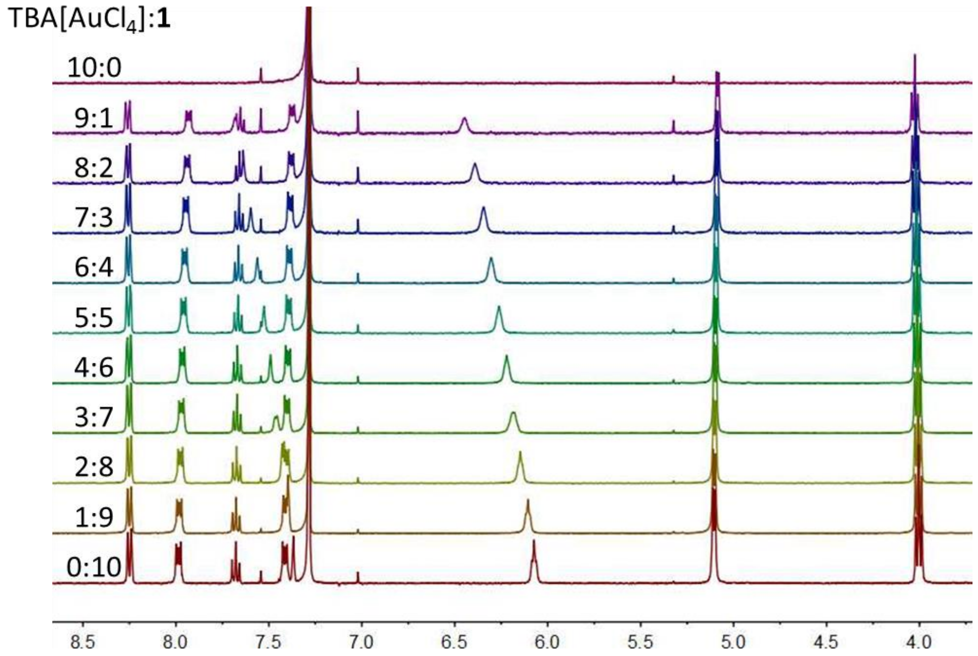

Figure S1: ^{1}H NMR spectra (500 MHz, $CDCl_3$, 298 K) by varying the ratio between **1** and TBA[$AuCl_4$] with a fixed total concentration. The total concentration of **1** and TBA[$AuCl_4$] is 1.0 mM and the starting concentration of **1** is 0.0 mM.

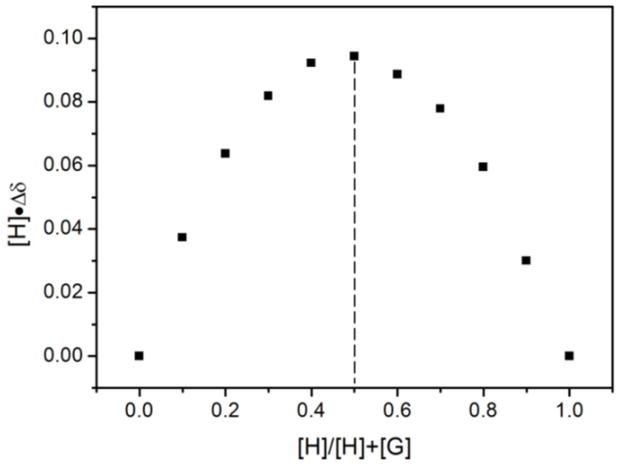

Figure S2: Job's plot constructed from the chemical shift change (Δδ) of the NH protons of **1** in ^{1}H NMR spectra in Figure S1 by varying the ratio between **1** and TBA[$AuCl_4$]. H represent the host **1** and G represent the guest TBA[$AuCl_4$].

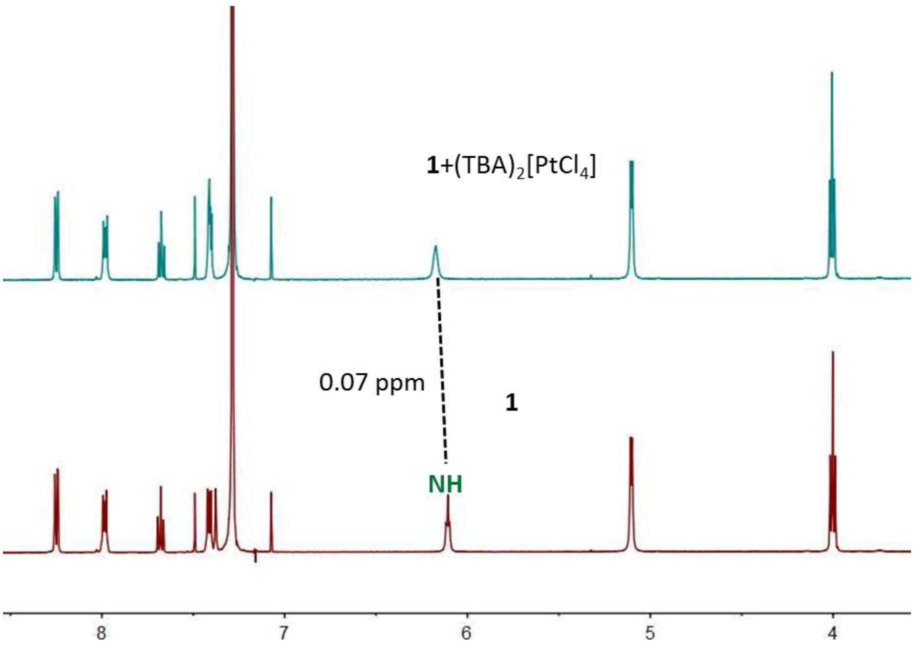

Figure S3: ^{1}H NMR spectra (500 MHz, $CDCl_3$, 298 K) of **1** (0.5 mM) and its equimolar mixture with $(TBA)_2[PtCl_4]$.

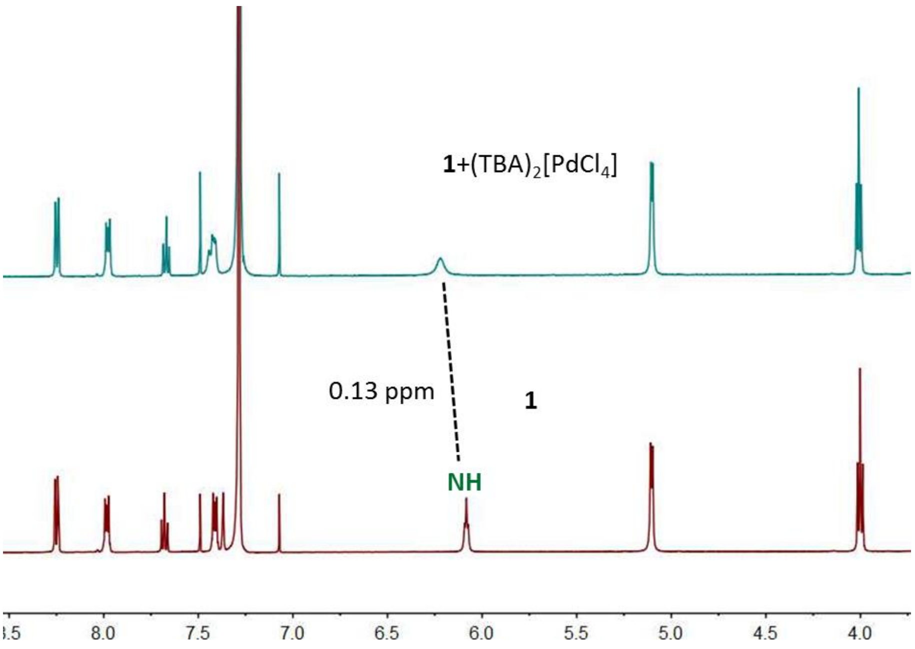

Figure S4: ^{1}H NMR spectra (500 MHz, $CDCl_3$, 298 K) of **1** (0.5 mM) and its equimolar mixture with $(TBA)_2[PdCl_4]$.

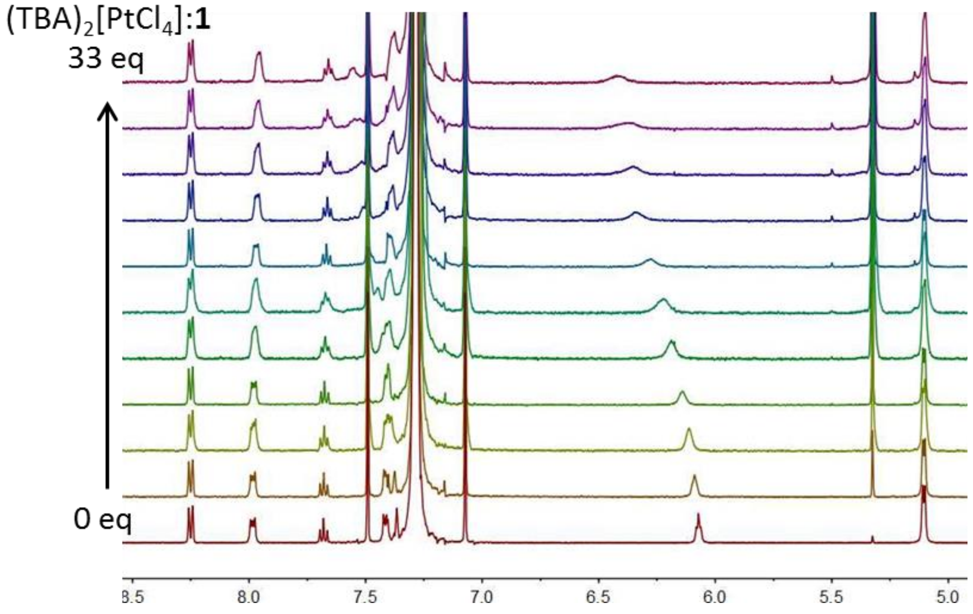

Figure S5: ^{1}H NMR spectra (500 MHz, $CDCl_3$, 298 K) by varying the ratio between **1** and $(TBA)_2[PtCl_4]$ with a fixed total concentration. The total concentration of **1** and $(TBA)_2[PtCl_4]$ is 1.0 mM and the starting concentration of **1** is 0.0 mM.

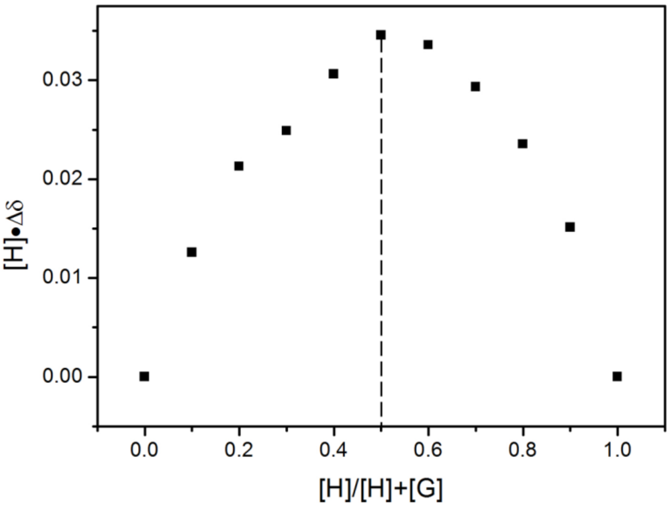

Figure S6: Job's plot constructed from the chemical shift change (Δδ) of the NH protons of **1** in ^{1}H NMR spectra in Figure S5 by varying the ratio between **1** and $(TBA)_2[PtCl_4]$. H represent the host **1** and G represent the guest $(TBA)_2[PtCl_4]$.

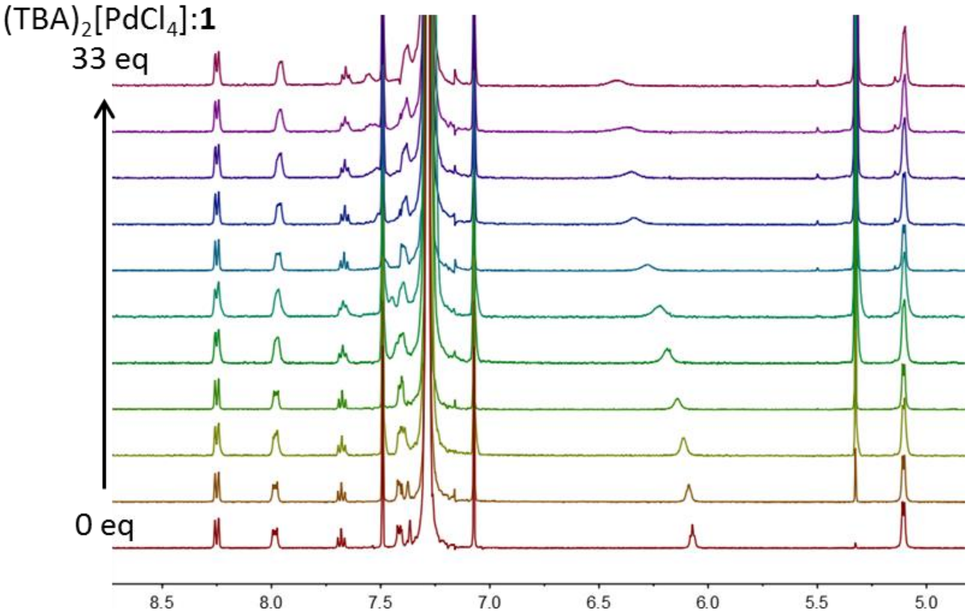

Figure S7: ^{1}H NMR spectra (500 MHz, $CDCl_3$, 298 K) by varying the ratio between **1** and $(TBA)_2[PdCl_4]$ with a fixed total concentration. The total concentration of **1** and $(TBA)_2[PdCl_4]$ is 1.0 mM and the starting concentration of **1** is 0.0 mM.

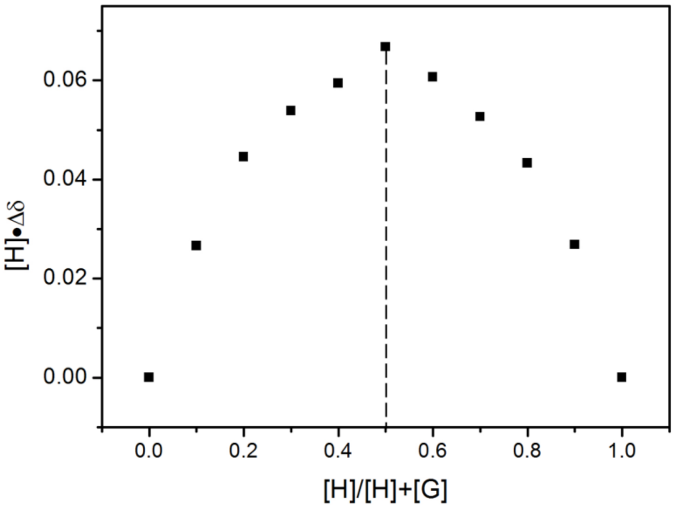

Figure S8: Job's plot constructed from the chemical shift change (Δδ) of the NH protons of **1** in ^{1}H NMR spectra in Figure S7 by varying the ratio between **1** and $(TBA)_2[PdCl_4]$. H represent the host **1** and G represent the guest $(TBA)_2[PdCl_4]$.

4. Binding constants determined by NMR titrations

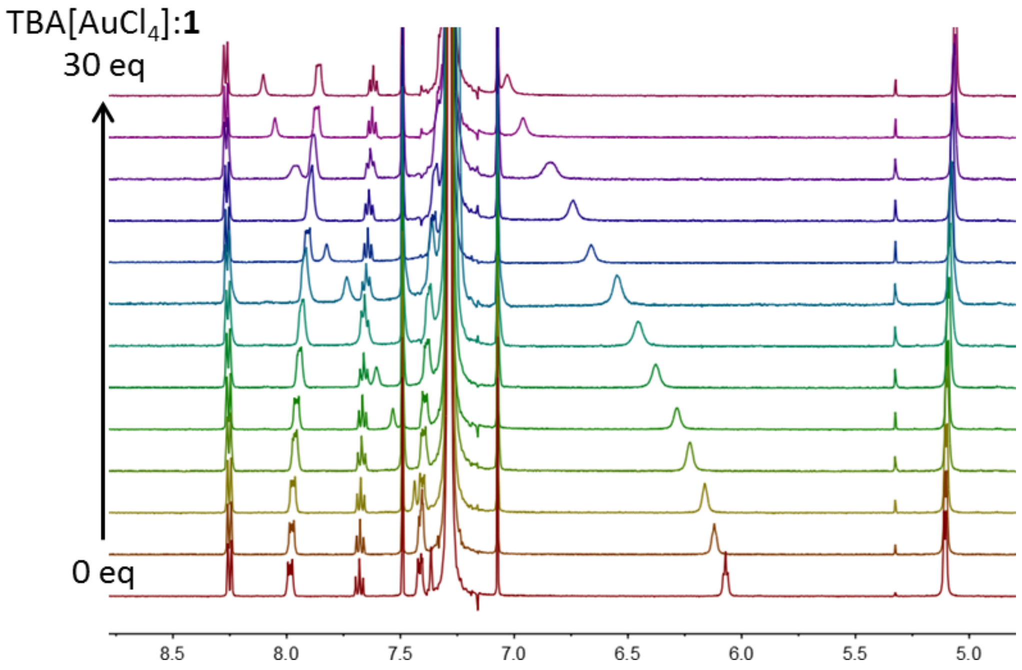

Figure S9: Partial ^{1}H NMR spectra (500 MHz, $CDCl_3$, 298 K) of **1** (0.25 mM) titrated with TBA[$AuCl_4$].

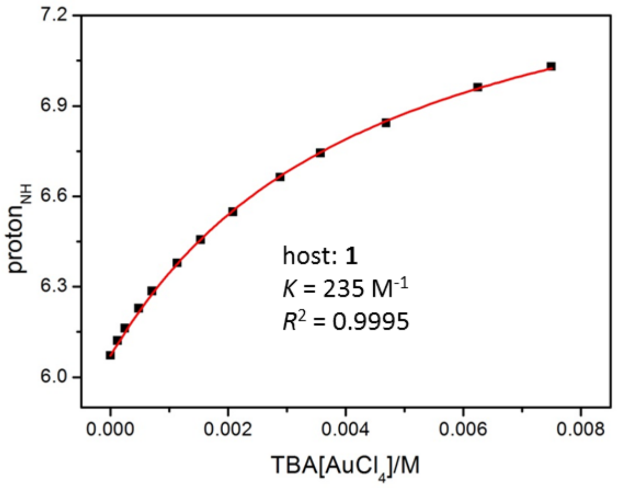

	K_a (M^{-1})	R^2
1st	235	0.9995
2nd	300	0.9988
3rd	260	0.9991
average	265 ± 33	

Figure S10: Non-linear curve-fitting for the complexation between **1** and TBA[$AuCl_4$] in $CDCl_3$ at 298 K.

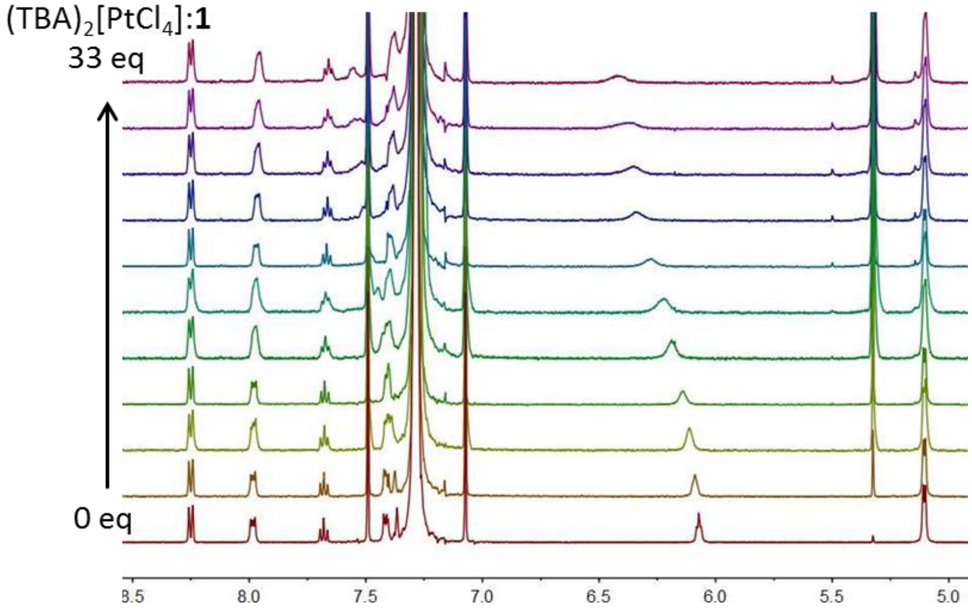

Figure S11: Partial ^{1}H NMR spectra (500 MHz, $CDCl_3$, 298 K) of **1** (0.25 mM) titrated with $(TBA)_2[PtCl_4]$.

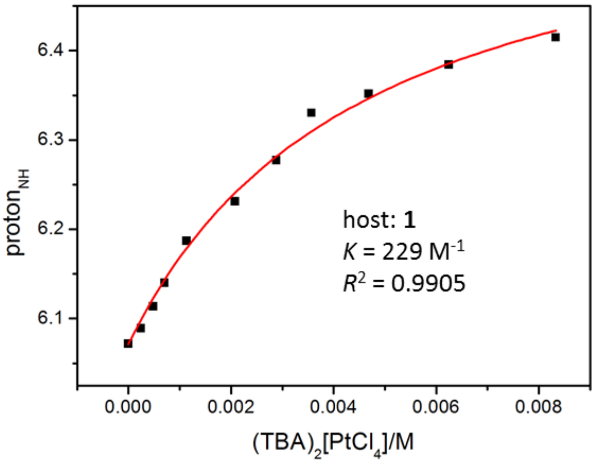

	K_a(M^{-1})	R^2
1st	229	0.9905
2nd	157	0.9913
3rd	181	0.9901
average	189 ± 36	

Figure S12: Non-linear curve-fitting for the complexation between **1** and $(TBA)_2[PtCl_4]$ in $CDCl_3$ at 298 K.

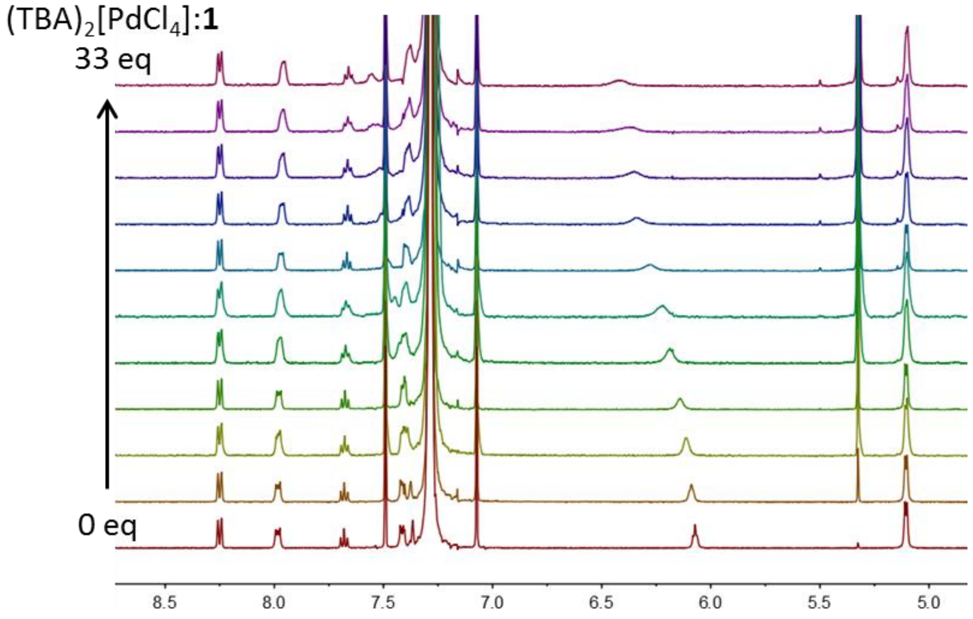

Figure S13: Partial ^{1}H NMR spectra (500 MHz, $CDCl_3$, 298 K) of **1** (0.25 mM) titrated with $(TBA)_2[PdCl_4]$.

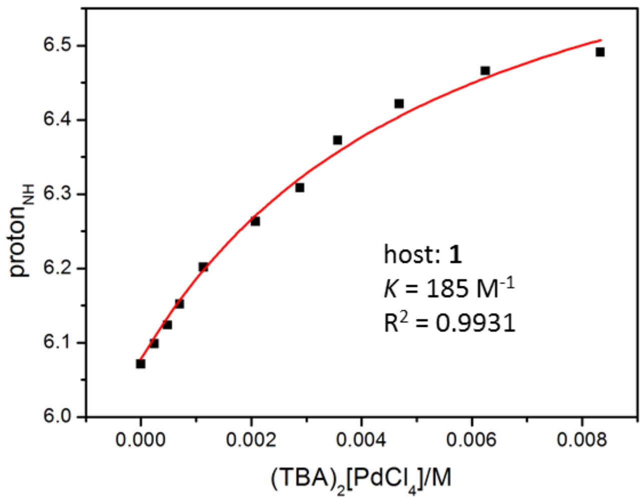

	K_a (M^{-1})	R^2
1st	185	0.9931
2nd	214	0.9936
3rd	194	0.9966
average	198 ± 15	

Figure S14: Non-linear curve-fitting for the complexation between **1** and $(TBA)_2[PdCl_4]$ in $CDCl_3$ at 298 K.

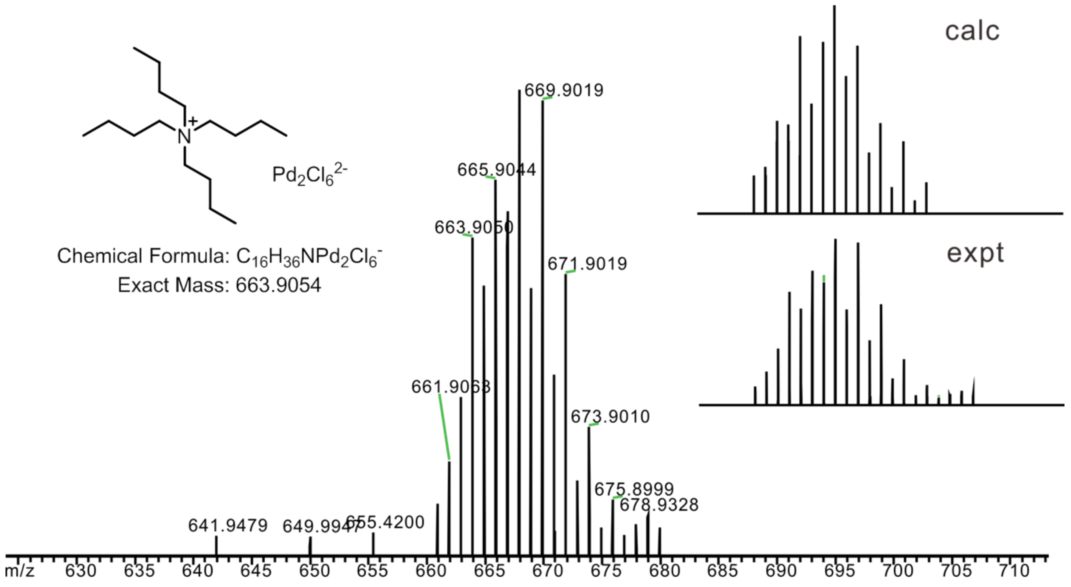

Figure S15: ESI mass spectrum of $(TBA)_2[PdCl_4]$. The peak of $TBA[Pd_2Cl_6]^-$ was found in the ESI mass spectrum of $(TBA)_2[PdCl_4]$.

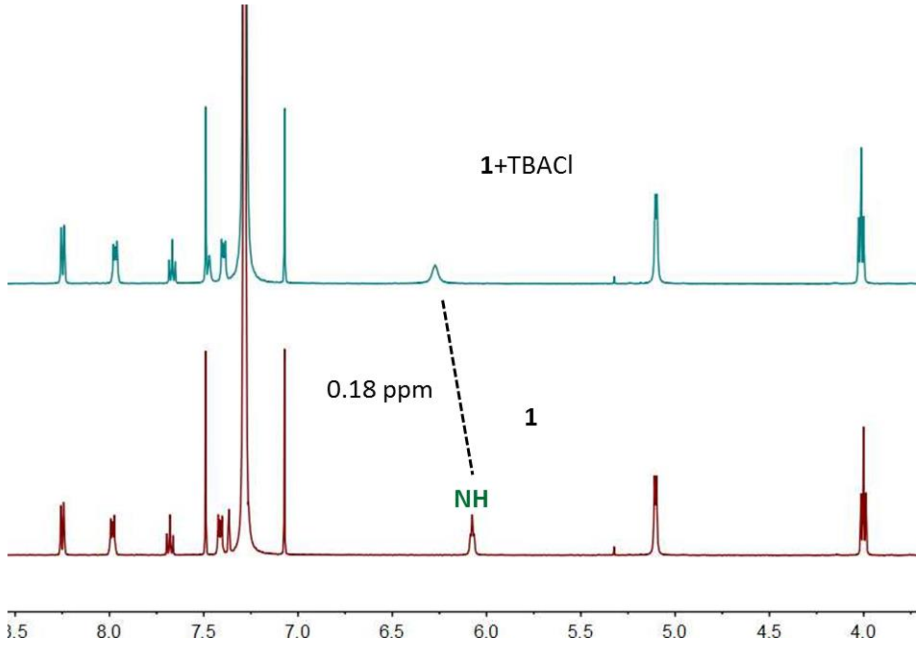

Figure S16: ^{1}H NMR spectra (500 MHz, $CDCl_3$, 298 K) of **1** (0.5 mM) and the equimolar mixture with TBACl.

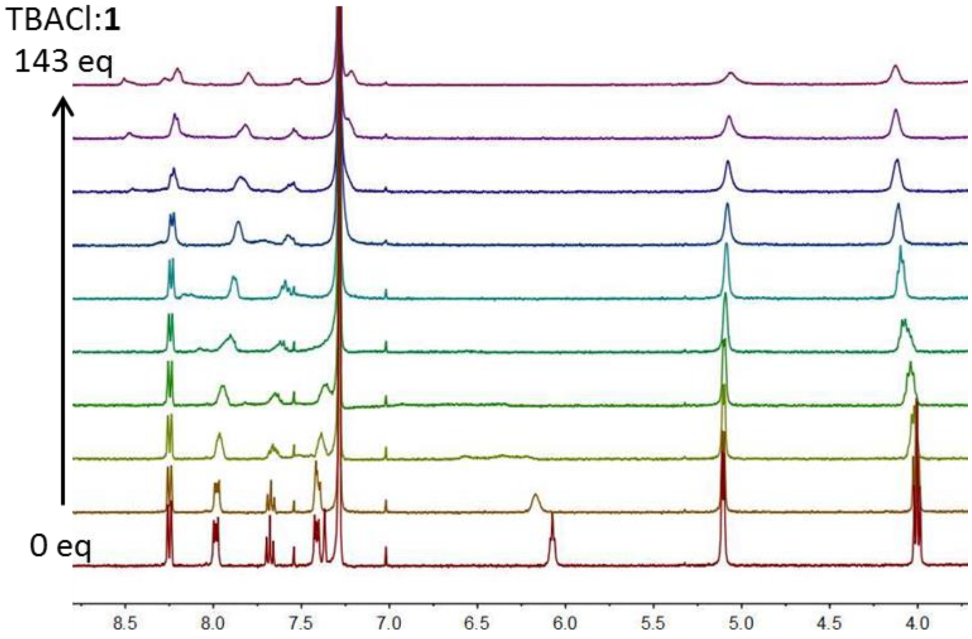

Figure S17: Partial ^{1}H NMR spectra (400 MHz, $CDCl_3$, 298 K) of **1** (0.5 mM) titrated with TBACl.

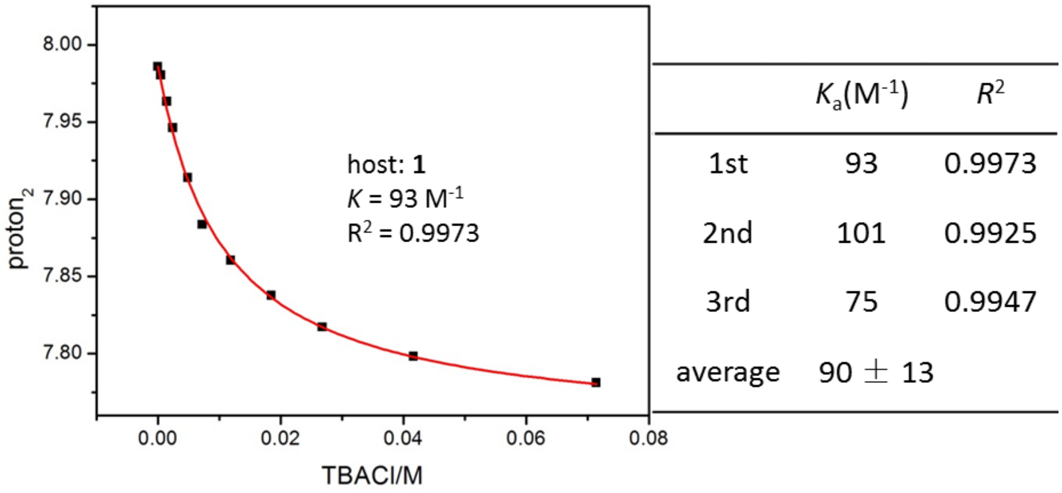

	K_a (M^{-1})	R^2
1st	93	0.9973
2nd	101	0.9925
3rd	75	0.9947
average	90 ± 13	

Figure S18: Non-linear curve-fitting for the complexation between **1** and TBACl in $CDCl_3$ at 298 K.

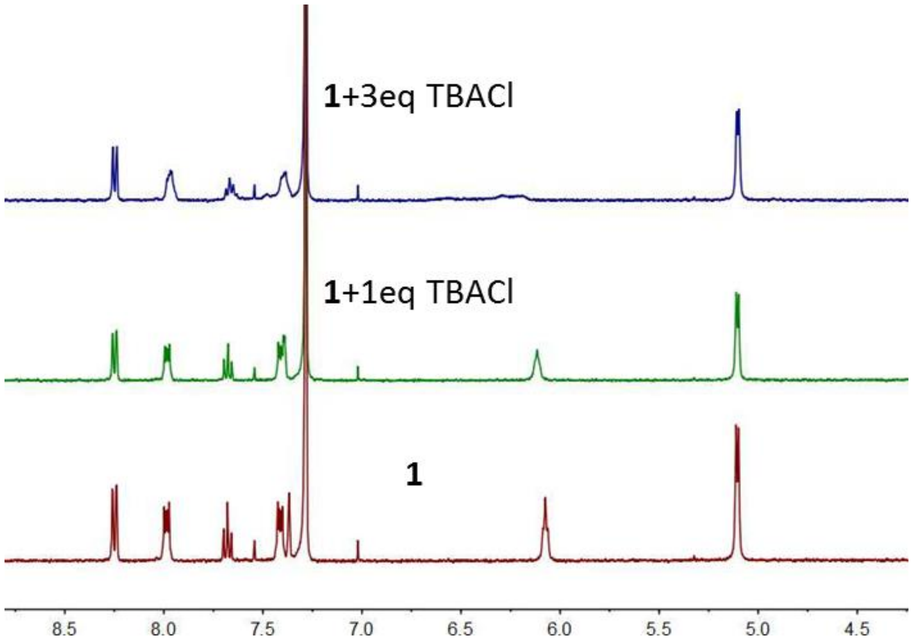

Figure S19: Partial ^{1}H NMR spectra (400 MHz, $CDCl_3$, 298 K) of **1** (0.5 mM) mixed with 1 equiv and 3 equiv TBACl.

5. ^{1}H NMR Spectra of 2 in the Presence of Guests

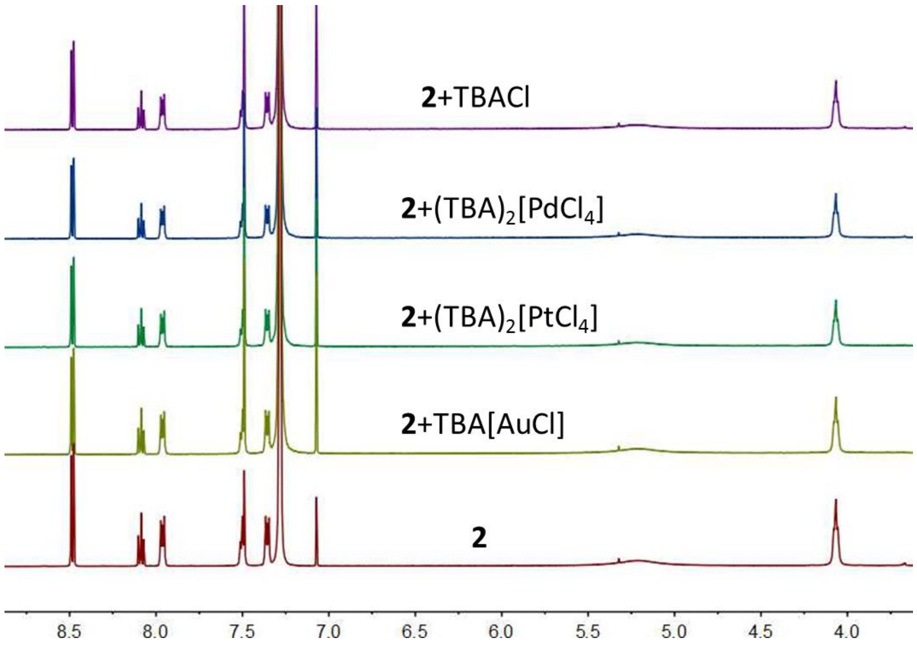

Figure S20: Partial ^{1}H NMR spectra (500 MHz, $CDCl_3$, 0.5 mM, 298 K) of **2** and its equimolar mixture with $TBA[AuCl_4]$, $(TBA)_2[PtCl_4]$, $(TBA)_2[PdCl_4]$, or TBACl.

6. Optical spectra of host–guest complexes

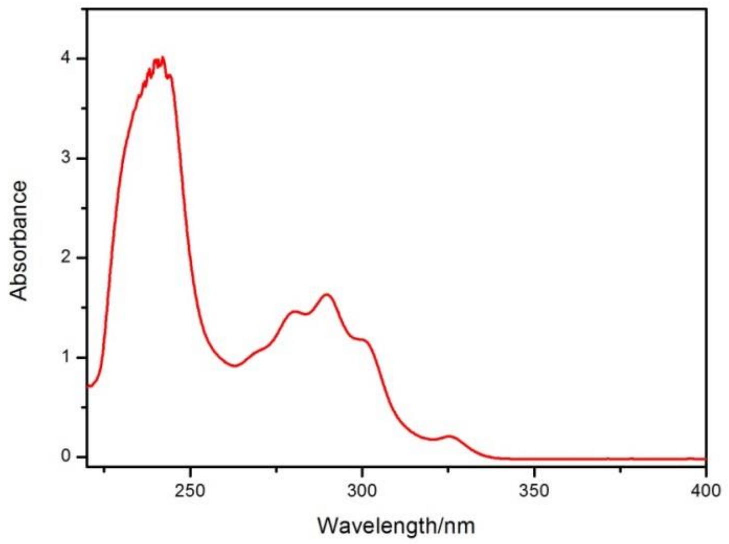

Figure S21: UV–vis absorption spectra of **1** in DCM at 298 K (0.1 mM).

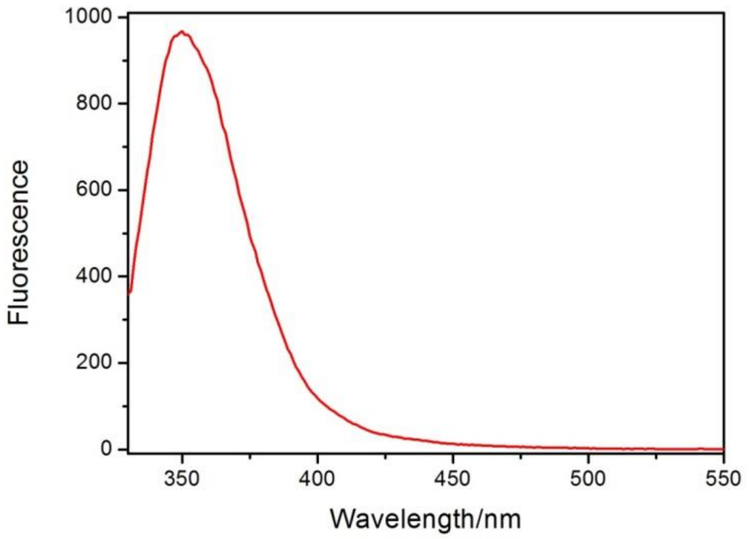

Figure S22: Fluorescence emission spectrum of **1** in DCM at 298 K (20 μM) with the excitation wavelength at 310 nm.

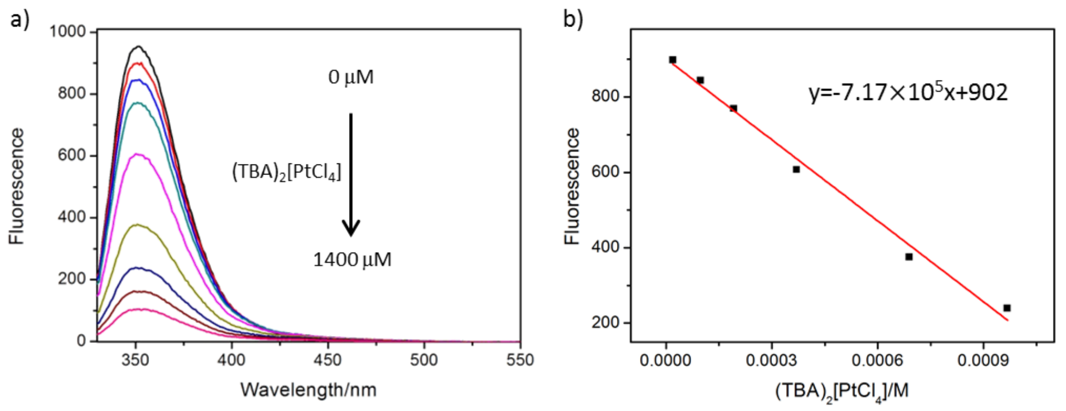

Figure S23: a) Fluorescence emission spectra of **1** in DCM at 298 K (20 µM) upon addition of different concentrations of $(TBA)_2[PtCl_4]$ (0–1400 µM) and then recorded in DCM at room temperature (λ_{ex} = 310 nm). b) Plot of fluorescence intensity versus the concentration of $(TBA)_2[PtCl_4]$ (20–970 µM).

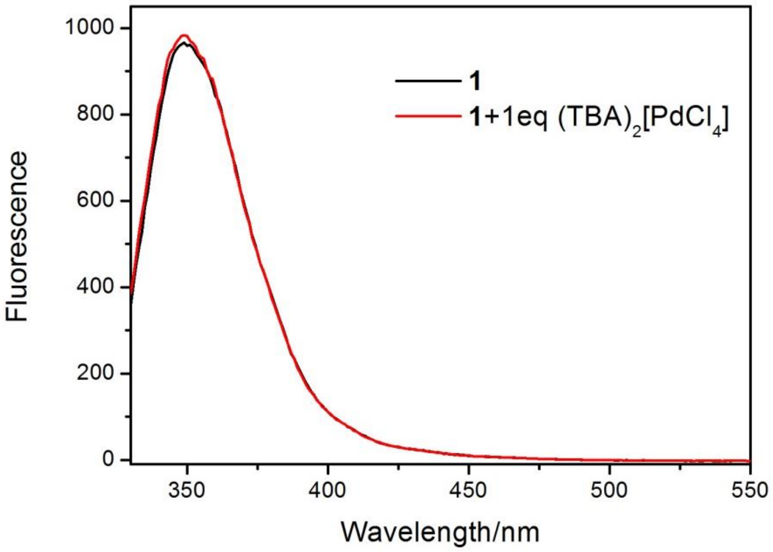

Figure S24: Fluorescence emission spectra of **1** in DCM at 298 K (20 µM) in the absence or presence of one equivalent of $(TBA)_2[PdCl_4]$.

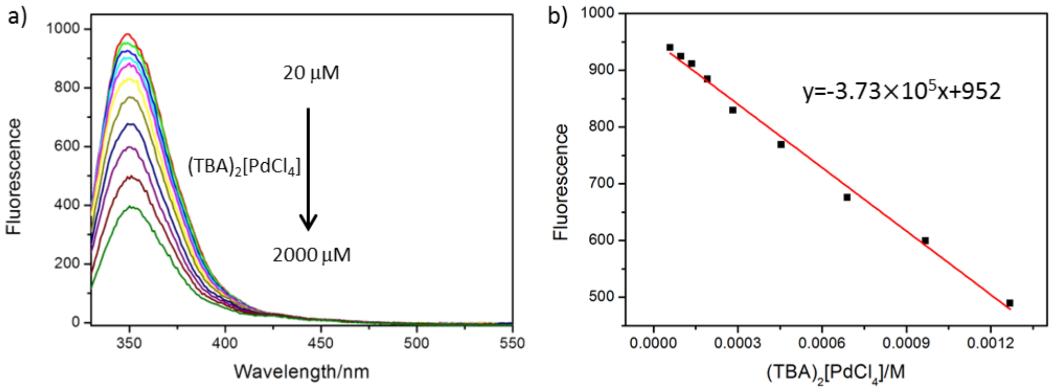

Figure S25: a) Fluorescence emission spectra of **1** in DCM at 298 K (20 μM) upon addition of different concentrations of $(TBA)_2[PdCl_4]$ (20–2000 μM) and then recorded in DCM at room temperature (λ_{ex} = 310 nm). The final concentration of **1** is 20 μM. b) Plot of fluorescence intensity versus the concentration of $(TBA)_2[PdCl_4]$ (60–1270 μM).

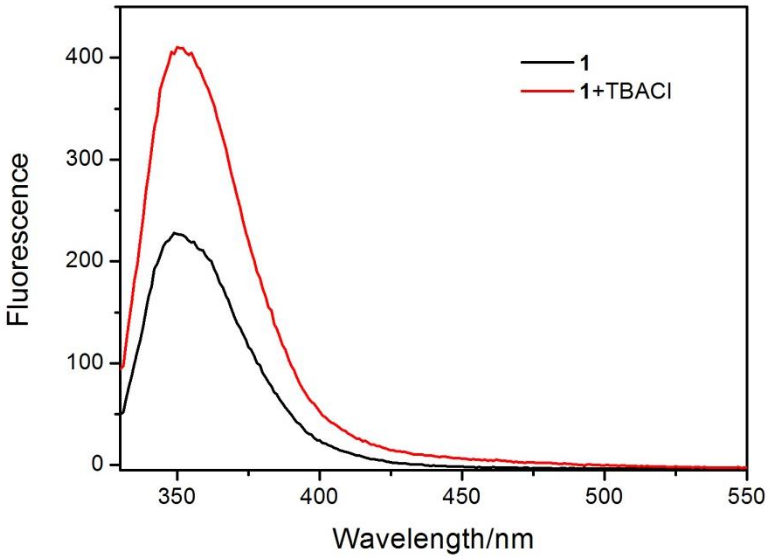

Figure S26: The fluorescence emission spectra of **1** in DCM at 298 K (5 μM) in the absence or presence of 10 equivalents of TBACl.

7. X-ray single crystal data

Crystal data of compound **1** was collected on a Bruker D8 VENTURE with Cu K_{α} radiation (λ = 1.5406 Å) at 173(2) K. The structures were solved by the direct method and different Fourier syntheses. All calculations were performed by full-matrix least-squares methods on F2 by using the SHELX-97 program [2,3], all non-hydrogen atoms were refined with anisotropic thermal parameters and the hydrogen atoms were fixed at calculated positions and refined by a riding mode. SQUEEZE routine implemented on PLATON was used to remove electron densities corresponding to disordered solvent molecules in the Crystal data.

Crystal data **1**: $C_{60}H_{72}N_6O_9$, M = 1021.23, triclinic, 0.3 × 0.2 × 0.1 mm, a = 13.0811(3) Å, b = 14.2610(3) Å, c = 15.5734(4) Å, α = 85.9530°(10), β = 86.3090°(10), γ = 69.0040°(10), V = 2703.17(11) Å3, Z = 2, 36373 reflections measured, 8005 independent reflections (R_{int} = 0.0283). Final R indices (I > 2σ(I)): R_1 = 0.0673, wR_2 = 0.1788, R indices (all data): R_1 = 0.0707, wR_2 = 0.1788. CCDC-1908471 contains the supplementary data for this structure.

8. References

1 Yang, L.-P.; Jia, F.; Zhou, Q.-H.; Pan, F. F.; Sun, J.-N.; Rissanen, K.; Chung, L. W.; Jiang, W. *Chem. Eur. J.* **2017,** 23, 1516 -1520. doi:10.1002/chem.201605701

2 Clark, R. C.; Reid, J. S. *Acta Cryst.* **1995,** A51, 887-897. doi:10.1107/S0108767395007367

3 Sheldrick, G. M. *Acta Crystallogr.* **2008,** A64, 112-122. doi:10.1107/S0108767307043930
